# Oral health care’s contribution to catastrophic spending in Canada: a descriptive study^☆^

**DOI:** 10.1016/j.hpopen.2025.100155

**Published:** 2025-11-20

**Authors:** Diego Proaño, Sara Allin, Beverley M. Essue, Sonica Singhal, Carlos Quiñonez

**Affiliations:** a Faculty of Dentistry, University of Toronto, Toronto, Ontario, Canada; b Institute of Health Policy, Management and Evaluation, Dalla Lana School of Public Health, University of Toronto, Toronto, ON, Canada; c North American Observatory on Health Systems and Policies, University of Toronto, Toronto, ON, Canada; d Public Health Ontario, Toronto, Ontario, Canada; e Schulich School of Medicine & Dentistry, University of Western Ontario, London, Ontario, Canada

**Keywords:** Catastrophic expenditure, Out of pocket payments, Financial protection, Universal health coverage, Dental health services

## Abstract

**Background:**

Oral health care (OHC) in Canada is largely financed through employer-sponsored insurance and out-of-pocket (OOP) payments and is generally excluded from its system of universal health coverage, although public financing will increase substantially with the introduction of the Canadian Dental Care Plan (CDCP). We generate estimates of catastrophic health expenditure (CHE) in Canada and assess the contribution of OOP spending in OHC on CHE between 2010 and 2019.

**Methods:**

We examined the Survey of Household Spending from 2010 to 2019 by year and in pooled cross-sections and followed the WHO/Europe methodology to determine CHE. Spending OOP in OHC was compared to medicines, medical products, outpatient care, diagnostic tests, and inpatient care. We assessed CHE and the share of OOP spending annually, nationally, provincially, across income quintiles and presence of private insurance including oral health coverage.

**Results:**

Estimates in CHE dropped from 5 % (2010) to 3.4 % (2019) and was more common among lower income groups, those without private insurance and Québec residents. Oral health care was the second highest contributor to CHE (after medicines) especially among the lowest income groups. Having private insurance yielded a higher share of OOP spending among lower than higher income groups.

**Conclusions:**

From 2010 to 2019, OOP spending in OHC was the second-highest contributor to CHE in Canada. Further monitoring is warranted to ensure financial protection is achieved for OHC after the full implementation of the CDCP.

## Highlights

• Catastrophic health expenditure in Canada dropped from 5 % in 2010 to 3.4 % in 2019.
• Oral health care was the second-highest contributor to catastrophic spending.
• Lower-income earners paid the highest out-of-pocket share on oral health care.
• Private insurance does not seem to financially protect income groups equally.

## 1 Introduction

Ensuring people who use health care, including oral health care (OHC), avoid undue financial hardship is a core goal in the United Nations’ Sustainable Development Goals and World Health Organization’s (WHO) universal health coverage agenda [1,2], as well as being a matter of social justice [3]. To achieve the WHO’s universal health coverage agenda for oral health, the WHO has included an indicator for financial hardship in OHC [2]. In this indicator, the WHO defines financial hardship as catastrophic health expenditure (CHE), which occurs when a household/person spends a large portion of their finances on paying for health care out-of-pocket (OOP) (i.e., direct payments, deductibles, co-payments) [4,5]. The WHO aims to monitor the “percentage of OOP payments dedicated to OHC among people incurring CHE” [2].

Canada’s case merits attention as OHC falls outside its system of universal health coverage. Payments OOP in the form of cost-sharing or direct payments play a central role in financing OHC in Canada [6], larger than in most other Organization for Economic Co-operation and Development (OECD) nations [7]. Though recently, Canada’s federal government has embarked on a series of reforms to expand OHC coverage for uninsured children, adults, and older adults from lower-to middle-income families. The Canada Dental Benefit was introduced in 2022 and was a direct cash transfer to families to pay for their children’s care. This benefit was replaced by the Canadian Dental Care Plan in late 2023, partially or fully covering the costs of some care for individuals and families whose incomes fall below $90,000. It is too early to evaluate the impact of these coverage expansions on financial protection, though historical estimates on OOP spending in OHC and CHE can provide a basis for future monitoring and evaluation.

Few studies have looked at the magnitude of OOP spending in OHC and its impact on CHE in Canada [[8], [9], [10]]. A 2009 study defined a large financial burden when the ratio of dental spending to the household’s total income surpassed 1 %, and found a high burden of dental spending among lower-income groups [8]. Two other studies defined CHE as health care OOP spending surpassing 10 % of the household’s total consumption (i.e., budget share) [9,10], and 40 % of the household’s net total consumption after deducting housing and food (i.e., capacity-to-pay) [10]. Medicines and OHC were found to be large contributors to CHE [9], mostly among those with higher-incomes [10]. However, the use of the budget share or capacity-to-pay estimated by deducting the household’s own spending has been criticized for potentially underestimating financial hardship among lower-income households and overestimating it among wealthier households [11].

As such, a new methodology to calculate CHE has been proposed by the WHO’s Regional Office for Europe, which establishes normative spending on food, rent, and utilities (i.e., needs-based line) that is deducted from the household’s total consumption. The needs-based line also reflects a poverty line to capture those impoverished and further impoverished by OOP spending [11]. This new methodology is regarded as equity-sensitive, adequate for high-income countries, and has been employed in a recent multi-country European study [12]. Such a methodology for CHE has not been used in Canada, which would allow for greater comparability with other high-income countries.

Our study aims to generate estimates of CHE in Canada and assess the contribution of OOP spending in OHC to CHE nationally and provincially between 2010 and 2019. Further, we estimate the annual trend of CHE across income quintiles and among those with private supplementary insurance that includes oral health coverage. Importantly, our study provides equity-relevant evidence aligned with the United Nation’s Sustainable Development Goals and WHO’s universal health coverage agenda for oral health [2], and provides policymakers with evidence of the magnitude of OOP spending in OHC, setting a baseline for future monitoring of Canada’s new federal initiatives.

## 2 Methods

### 2.1 Data source

We used pooled cross-sectional data from Statistics Canada’s Survey of Household Spending (SHS) from 2010 to 2017 and 2019, which is nationally and provincially representative. We chose to analyze the years 2010–2019, as it allows us to contextualize our findings to a pre-pandemic health care system and the current pre-dental care coverage expansion. Although the impact of the CDCP in 2023 would have been relevant to analyze, the SHS 2023 dataset was not available at the time of this study. The SHS collects detailed data on household consumption, which are annualized to adjust for the use of different recall periods in the questionnaire. Statistics Canada employs a multi-stage sampling frame and selected households are invited for face-to-face interviews. Since the survey methodology for Canada’s territories has not been consistent over the 2010–2019 years and because provincial data has been used for nationally representative estimates, our study only uses the provincial sample (Table S1).

The SHS excludes institutionalized residents, members of the Canadian Armed Forces living in military camps, and households on Indigenous reserves, which comprise about 2 % of the target population. The few data collection issue exclusions (<0.5 % of the target population), are adjusted for in the sampling weights [13].

Between 2010 and 2019, a total of 159,945 households were targeted for SHS interviews with a response rate of around 65 %. In total, 104,254 households were interviewed from 2010 to 2019 (Table S2). From our eligible interview sample, we decided to exclude households reporting a negative total consumption or no food expenditure (n = 329), achieving a final sample of 103,925 households.

We exclusively analyzed the SHS interview data. Although the SHS also collects diary data, where food spending information is more comprehensive compared to the one food questionnaire item from the SHS interview, using the SHS diary data would have reduced our sample size considerably (between 50 % to 70 %) (Table S2). Further, the income source used as part of the weight calibration of the interview and the diary also differs [13].

### 2.2 Household’s financial resources, health care out-of-pocket spending, and private insurance

Household total consumption was calculated from different spending items, which have slightly changed throughout the survey years. We examined modifications to the questionnaire’s spending items across the SHS years (2010–2017) to create comparable spending groups matching SHS 2019. Household spending items employed in our analysis are organized according to Classification of Individual Consumption According to Purpose (COICOP) 2018 (File S1). In terms of household income, we used a before-tax household income, which included employment income, government transfers, retirement pensions, investment income, and other sources of income (e.g., scholarships, support payments, etc.) for all SHS years.

Health care OOP spending included directs costs for OHC, medicines, medical products, outpatient care, diagnostic tests and other services, and inpatient and residential care (see Table S3). The SHS used a 12-month recall period for all health care services, except for prescribed medicines (3 months). Out-of-pocket payments included direct costs, deductibles and co-payment requirements and excluded OOP spending reimbursed or to be reimbursed by any third party (e.g., government or private insurance). We determined having private supplementary insurance including oral health coverage based on whether households had made any payments (e.g., premiums) to either private health or oral health insurance.

### 2.3 Catastrophic health expenditure

We focus our measurement of CHE using the equity-sensitive capacity-to-pay methodology formulated by the WHO/Europe and accompany our results with the commonly employed budget share approach (see supplementary material) [11,14,15]. In CHE estimation, a household’s health care OOP spending is measured against the household’s financial resources and if the spending is above a certain threshold, the spending is considered catastrophic. In capacity-to-pay measures, the household’s financial resource is defined as the net budget after subtracting an allowance for subsistence needs [4].

What is unique about the WHO/Europe methodology is that it defines capacity-to-pay as the household’s total annual consumption minus a normative spending on food, rent (housing), and utilities (e.g., water, electricity, gas) [11,15] (i.e., a basic needs line). For each SHS year, we calculated– using survey weights– the basic needs line at the national level by averaging consumption per equivalent adult (using the OECD equivalence scale) on food, rent, and utilities among households ranked between the 25th and 35th percentile of total consumption per equivalent adult. The obtained estimate is adjusted to the household level accounting for household size and composition [11].

In the WHO/Europe capacity-to-pay measure, some households may have or end up with a negative capacity-to-pay, as their household’s budget may be lower before or after subtracting the basic needs line. For those households, any health care OOP spending is considered catastrophic [11]. Importantly, this methodology captures households who experience impoverishing (i.e., the household’s capacity-to-pay was lower than the basic needs line after subtracting) and further impoverishing (i.e., the household’s capacity-to-pay was lower than the basic needs line before subtracting) OOP spending. Further, we also examined the risk of impoverishment as 120 % above the basic needs line [16].

Thresholds to determine CHE were set at 40 % of capacity-to-pay, as commonly reported in the literature [14]. We also explored the impact of lowering the capacity-to-pay threshold to 10 %. Importantly, the WHO/Europe approach is equity-sensitive, as the effective threshold households need to surpass to become catastrophic is higher for higher-income than lower-income earners [11].

### 2.4 Descriptive analysis

We employed survey weights corresponding to each SHS year to provide precise national and provincial estimates, and we accompanied our estimates with a 95 % confidence interval, except for the share of OOP spending, and we describe significant differences when confidence intervals are not overlapping. To describe the trend in CHE in Canada, we used the SHS cross-sectional years to estimate the proportion of households (and members) facing CHE. For the provincial-level estimates, however, we have employed a pooled year analysis of the SHS (2010–2019).

Our findings focus on the WHO/Europe 40 % capacity-to-pay, CHE estimates using the other CHE methods are found in the supplementary material. We also describe the trend of impoverishment, further impoverishment, at risk/not at risk for impoverishment, and those not making any OOP spending in Canada and the provinces between the 2010–2019 period. We employed the household size and composition equivalized version of income for the distribution across income quintiles.

To describe the contribution of OOP spending in OHC to CHE, we estimate the share of OOP spending made to each health care service (OHC, medicines, outpatient, medical products, diagnostic tests and other services, and inpatient and residential care) among the general population (nationally and provincially) and those facing CHE. Among those facing CHE, we examine the distribution across income quintiles. At the national and provincial levels, we employ a pooled-year analysis (2010–2019) and at the national level we complement our analysis with a split-period analysis (2010–2015; 2016–2019), reflecting a change in Canada’s federal government.

Further, we stratified our annual national estimates and the share of OOP spending nationally and provincially by private insurance (with/without) and examine the distribution across income quintiles. We assess the provincial-level estimates CHE among those with and without private insurance. We also complement our descriptive analysis with the proportion of households facing CHE nationally, provincially, by private insurance, and across income quintiles for 2010–2019 as a pooled-year analysis.

## 3 Results

### 3.1 Catastrophic health spending estimates

**Fig. 1:**
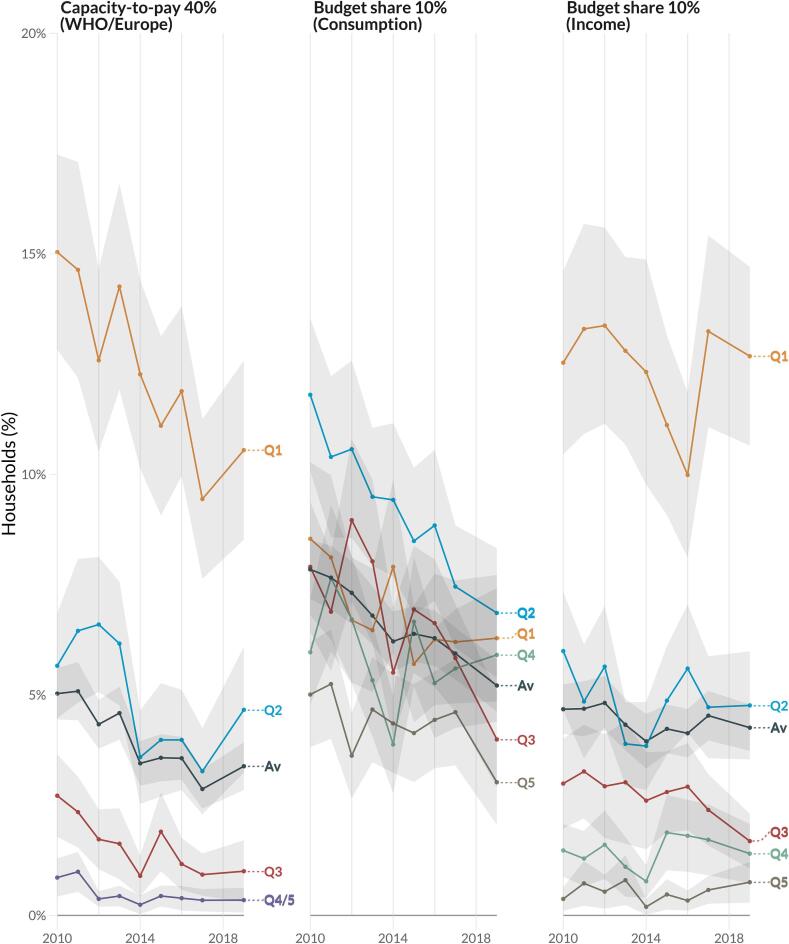
Proportion of households in Canada experiencing catastrophic health expenditure using capacity-to-pay and budget-share methods across income quintiles from 2010 to 2019. Capacity-to-pay defines household resources as total consumption net from normative food, rent, and utilities spending developed by the WHO Regional Office for Europe (WHO/Europe); basic share approach defines household resources as either consumption or income. All catastrophic health expenditure thresholds are represented in % next to the capacity-to-pay and basic share methodology employed. Shaded area represents 95 % confidence interval. Income quintiles (Q) ordered from lowest (Q1) to highest (Q5), Q4 & Q5 have been merged in capacity-to-pay 40 % approach for privacy risk reasons. Av, represents average.

Between 2010 and 2019, the proportion of households facing CHE declined (Fig. 1). At the same time, private supplementary insurance including oral health coverage increased from 29.9 % (95 % CI:28.7 %—31.0 %) to 49.4 % (95 % CI:48.0 %—50.9 %) (Table S4). In Fig. 1, the WHO/Europe 40 % capacity-to-pay metric displays a significant drop from 5 % (95 % CI: 4.4 %–5.6 %) in 2010 to 3.4 % (95 % CI: 2.8 %–3.9 %) in 2019. In absolute terms, around 0.5–0.8 million households (1.1–1.8 million people) face catastrophic spending each year (Table S5). We also explored the impact of lowering the capacity-to-pay threshold to 10 %, which increases the proportion of households (and people) facing CHE by around 4–5 times (Table S5).

Further, we found a decrease in the proportion of households (and members) spending OOP over time and a slight decrease in those impoverished, further impoverished, and those at risk of impoverishment (Table S6).

CHE varied across income quintiles: the lowest and to some extent the second-to-lowest income quintile experience a higher proportion of CHE than the average household population in Canada (Fig. 1). We also found a generally lower CHE estimate among those with private supplementary insurance, including oral health coverage, than those without; however, among those with private insurance, lower income groups had a higher estimate of CHE than the average, and those in the higher income groups consistently experienced lower CHE. It also seems CHE estimates for both insurance groups are dropping (File S2).

**Fig. 2:**
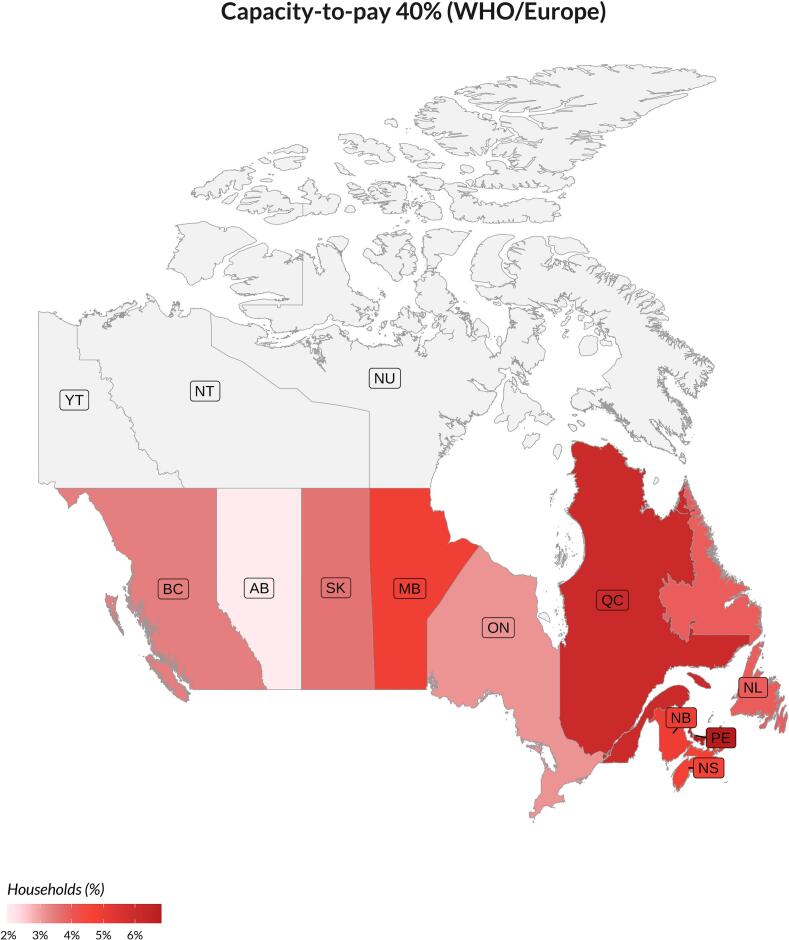
Catastrophic health expenditure in Canada by provinces from 2010 to 2019. Capacity-to-pay defines household resources as total consumption net from normative food, rent, and utilities spending developed by the WHO Regional Office for Europe (WHO/Europe), using a 40 %. BC, British Columbia; AB, Alberta; SK, Saskatchewan; MB, Manitoba; ON, Ontario; QC, Québec; NB, New Brunswick; NL, Newfoundland & Labrador; PE, Prince Edward Island; NS, Nova Scotia. We don’t have data available for YT, Yukon; NT, Northwest Territories; and NU, Nunavut, the three territories in Canada.

Across the Canadian provinces, we found Ontario and Alberta to have the lowest CHE estimates, while Québec and Prince Edward Island had the highest (Fig. 2 and Fig. S1). We also observe the highest share of OOP spending in OHC in Québec and the least in Newfoundland & Labrador and Prince Edward Island, which was consistent across CHE methodologies (Fig. S2).

### 3.2 The contribution of oral health care to catastrophic health spending

Around half of the household population consistently paid OOP for OHC during 2010–2019 (Table S4) and 1.8 million households (3 %) faced CHE — affecting mostly households in the lower income quintile (Table S7).

The number of households spending OOP for OHC is the second highest (around 6.9 million per year), the highest being for medicines (around 8.8 million per year), which is also reflected across income quintiles. In the second quintile, about half more households spending on OHC than the lowest quintile, which is the largest difference compared to other health care services. Among households facing CHE, we find a positive gradient in the proportion of households spending on OHC across income quintiles (Table S7).

**Fig. 3:**
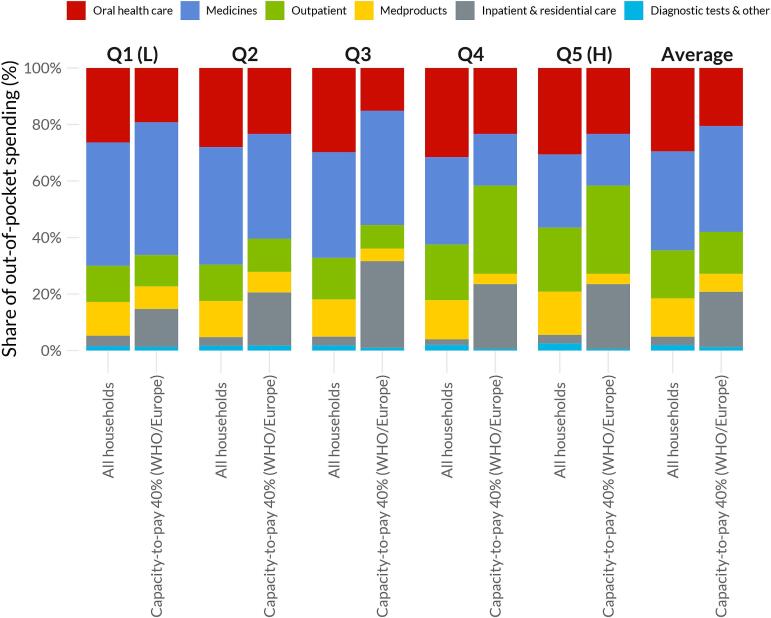
Share of health care out-of-pocket spending by income quintiles among households in Canada and those experiencing catastrophic health expenditure Capacity-to-pay defines household resources as total consumption net from normative food, rent, and utilities spending developed by the WHO Regional Office for Europe (WHO/Europe), using a 40 %. Income quintiles (Q) ordered from lowest (L) to highest (H). Inpatient included hospital care, nursing homes and other residential facilities; diagnostic tests and other services included laboratory services, rental of medical equipment, ambulances, as well as weight control and quit-smoking programs.

Oral health care was the second most important contributor (after medicines) to CHE in terms of OOP spending, especially across the lowest income quintiles (Q1:19.2 %; Q2: 23.3 %). Among those facing CHE, OHC comprised around two of every ten OOP dollars spent on health care (Fig. 3 and Fig. S3). Using a split-period comparison, we find an increase in the share of OOP spending in OHC among the lowest quintiles in 2016–2019 compared to 2010–2015 (Fig. S4).

**Fig. 4:**
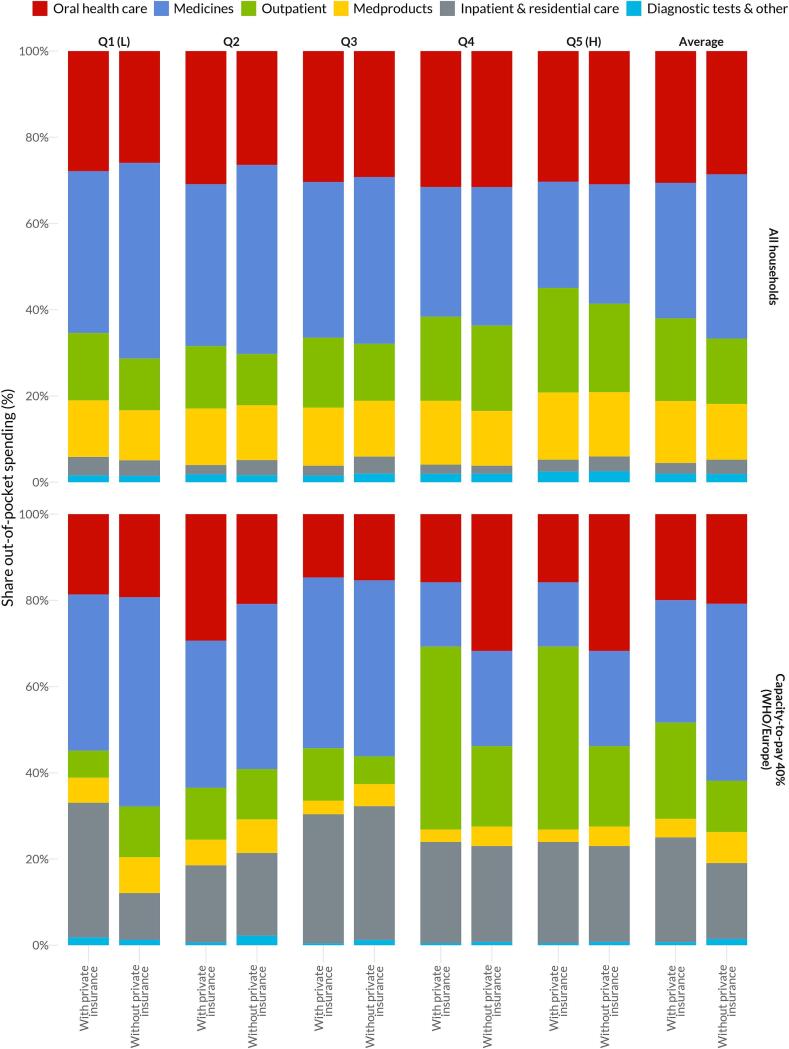
Share out-of-pocket spending among households in Canada with and without private supplementary insurance including oral health coverage Capacity-to-pay defines household resources as total consumption net from normative food, rent, and utilities spending developed by the WHO Regional Office for Europe (WHO/Europe), using a 40 %. Income quintiles (Q) ordered from lowest (L) to highest (H). Inpatient included hospital care, nursing homes and other residential facilities; diagnostic tests and other services included laboratory services, rental of medical equipment, ambulances, as well as weight control and quit-smoking programs.

The relationship between private insurance and OHC spending varies according to the income group. For lower income quintiles, those with private insurance have a higher share of OOP expenditure in OHC, whereas for highest income quintile, private insurance is associated with lower OOP expenditure in OHC, suggesting more comprehensive coverage for these higher income households (Fig. 4, Fig. S5, and S6).

## 4 Discussion

Across all Canadian households, we found CHE decreased from 2010 to 2019, with those in lower income quintiles consistently experiencing a significantly higher proportion of CHE. We found that medicines and OHC are the highest and second-highest contributors to CHE, respectively. Our results provide a pre-pandemic baseline for Canada in the context of monitoring and evaluating its new federal OHC coverage initiatives. Similarly, our results provide a baseline for monitoring financial protection goals in universal health care coverage as described in the United Nations’ Sustainable Development Goals [17] and reflected in the WHO’s Global Oral Health Action Plan [2]. To monitor CHE as outlined in the WHO’s Global Oral Health Action Plan [18] and to provide international comparability, we employed the equity-sensitive metric of CHE proposed by the WHO’s Regional Office for Europe [11]. We also found that our results are consistent across different CHE methodologies (i.e., budget share or lowering the capacity-to-pay threshold to 10 %). Ultimately, contextual and system-level factors need to be considered when interpreting our findings.

### 4.1 Catastrophic health expenditure in Canada

Not all drops in catastrophic spending are desirable, as two possible conflicting reasons explain this: either more people are being financially protected from catastrophic costs or more people are simply financially unable to enter the health system and access care. For example, the lowest estimate in catastrophic spending happened during 2014/2015, which coincides with an increase in living costs in Canada [19] and the lowest proportion of households spending OOP. This means fewer people may have experienced CHE because they simply could not pay for health care services. At the same time, the increase in private supplementary insurance coverage including OHC may have had a positive role in decreasing CHE, although unequally across income groups. In fact, if we examine the contribution of impoverishment and further impoverishment to CHE, we see an increase during the 2010–2019 period, which suggests little improvement in financial protection among lower-income groups despite a decrease in CHE. In short, the decrease of CHE in Canada arguably relates to a mix of higher financial protection from private health insurance and the experience of higher cost-barriers to care, the former likely disproportionately benefitting higher income groups and the latter likely disproportionately affecting lower income groups.

### 4.2 Comparing catastrophic health spending in Canada and Europe

Canada’s CHE 2019 estimate (3.4 %) would rank 13th among 29 other high-income nations in Europe, 16th for impoverishment (0.4 %), and 22nd for further impoverishment (2.4 %) [12]. In all these cases, Canada consistently ranks below countries like the Netherlands, Germany, and the UK [12], countries that have levels of public and private financing for OHC, helping them achieve universal or near universal coverage for their populations. Impoverishment and further impoverishment in Canada due to health care OOP spending also explains around three-quarters of CHE, which is the second-to-highest contribution compared to any other high-income nation in Europe [12]. Again, these estimates highlight concerning inequity in Canada: those unable or barely able to meet basic needs incur health care OOP spending (including OHC), and, as a result, lower-income households face greater financial hardship due to such spending than those in higher-income households. The extent to which Canada’s CDCP will fill the gaps and helps to ensure financial protection among those most in need of OHC remains to be seen.

Compared to Commonwealth and other select nations, during our study period, Canada ranked second-to-highest in cost-barriers to OHC, while the Netherlands had the lowest such cost-barriers along with the UK and Germany [20]. Cost-barriers to OHC are a recurring theme in Canada, which are disproportionality greater among lower-income than higher-income households [[21], [22], [23], [24], [25]], restricting those most in need from accessing OHC services. To limit the role of OOP spending in OHC, the Netherlands, for instance, provides statutory coverage for OHC for all children up to the age of 21, limited services for older adults, and regulates dental pricing maximums through its National Health Authority [26]. Limiting OOP spending in OHC suggests greater financial protection in the Netherlands than in Canada.

### 4.3 The contribution of oral health care to catastrophic spending: The Canadian context

It is not a surprise to find medicines and OHC as top contributors to CHE in Canada, as these are excluded from the country’s system of universal health coverage (also known as Medicare) [27]. It should be noted that surgical OHC and medicines that requires a hospital setting for medical reasons is generally covered as part of Medicare. As well, the federal government covers OHC treatments for Indigenous populations as part of their fiduciary relationship with Indigenous Peoples [28]. For all other dental procedures – which are overwhelmingly provided in privately owned offices – there is scarce public coverage (around 6 % of total OHC expenditure) [6,29], placing Canada among the lowest public funders for OHC among OECD countries [7]. At the provincial and territorial level, each publicly financed OHC program varies in what and how much is covered. Existing provincial and territorial programs generally target children from the lowest income households and, to a lesser extent, lower-income adults and older adults [29,30], which may explain why the second-to-lowest income earners facing CHE paid a higher share of OOP on OHC than the lowest income earners.

We also observed an increase in the proportion of households with private insurance including oral health coverage throughout 2010–2019, but this does not seem to be financially protecting those in the lower income groups. In Canada, private health insurance through employee-benefit plans is the main pathway by which residents gain coverage for OHC services [28]. There is relatively robust private health insurance coverage through employee benefits packages for middle- to high-income groups [28,29]. Patients are typically not expected to pay all OHC costs upfront, as dentists (through contractual arrangements) often have private insurers pay them directly on the patient’s behalf. Yet across Canada, there are many instances where the patient does pay some or all costs upfront, whether because of a copayment or because the dentist prefers that all costs be paid directly by the patient. In the latter, the dentist most often submits a claim to the insurer on the patient’s behalf and then the insurer reimburses the patient directly [31]. The quality of employee benefits for OHC has, however, decreased over time and higher-paid jobs are more likely to possess a more comprehensive OHC package of services [28]. There is also no obligation for employers to provide coverage for OHC services, and working low-income households are generally facing greater oral health needs and OHC barriers – especially cost-related [28].

The high CHE estimates found in Québec and Prince Edward Island are arguably related to different causes. Although both provinces displayed the highest CHE estimates, the share OOP spending in OHC among those experiencing CHE was highest in Québec and lowest in Prince Edward Island. Québec’s low proportion of private health insurance [32] means the public system has a greater role in providing financial protection, but it is said to be underutilized [33,34]. As a result, Québec arguably shows a higher reliance on OOP spending for OHC. On the other hand, Prince Edward Island had the lowest income per capita across the provinces throughout the study period [35]. As our basic needs line was constructed at the national level, the high CHE estimate in Prince Edward Island may reflect a generally lower capacity-to-pay.

### 4.4 Study limitations

Our study has limitations that must be considered. First, it is a descriptive study using cross-sectional survey data. Although a longitudinal design would have been desirable, our estimates still provide nationally and provincially representative granular information. Second, our study does not provide a window into the different types of OHC services paid OOP and lacks information on oral health needs. Although analyzing individual types of OHC services is relevant, we believe the accrued amount of OOP spending on OHC services provides insight into the financial hardship people face for OHC services as a whole. Third, CHE metrics exclude those who forgo OHC because of cost, so we have considered cost-barriers to OHC when interpreting our findings. Fourth, although the use of the SHS diary’s detailed food consumption would have been desirable, we would have lost between 50–70 % of our sample. Exclusively using the interview content did not seem to affect our results, as our CHE consumption-based budget share approach estimates are comparable to other studies in Canada [9,10]. Fifth, we captured private insurance including oral health coverage by assessing if the household had reported any premium payments, which seems to underestimate the true number of households with private health insurance [32]. Our estimates, however, are still valuable as they capture the negative gradient of private health insurance across income quintiles as reported in the literature [22,36].

### 4.5 Policy implications and further research

Our study is the first to employ the WHO/Europe CHE methodology in Canada, capturing those being impoverished and further impoverished from OOP spending. Also, our study aligns with the universal health coverage goal of financial protection, which has been highlighted in the WHO’s Global Oral Health Action Plan [2]. Further, our study provides pre-pandemic information on the contribution of OHC to CHE, which can be used to monitor Canada’s new investments in OHC coverage, namely the CDCP [34]. Further research is also needed to fully understand the impact of OOP spending in OHC on financial hardship (i.e., CHE and impoverishment), particularly what OHC services specifically lead to financial hardship, as that can inform what and how specific OHC services could be covered under a universal health coverage agenda.

## 5 Conclusion

Between 2010 and 2019, Canada’s health care (and OHC) system(s) seem to be providing some level of financial protection to the general population, but lower-income households still disproportionately face higher levels of CHE. Given that OHC is excluded from Canada’s system of universal health coverage, paying OOP for OHC is the second-highest contributor to CHE. Although private health insurance including oral health coverage provides some level of financial protection, it does not protect income groups equally, where the lowest income earners pay a higher share of OOP for OHC than those with higher incomes. If Canada’s goal is equity in financial protection, there is a major challenge ahead. Importantly, our findings provide a pre-pandemic baseline to monitor CHE, the contribution of OOP spending in OHC on CHE, as well as the impact of Canada’s new federal investments in OHC, which aim to provide financial protection and address the equity concerns raised.

## CRediT authorship contribution statement

**Diego Proaño:** Writing – review & editing, Writing – original draft, Visualization, Validation, Software, Methodology, Investigation, Formal analysis, Data curation, Conceptualization. **Sara Allin:** Writing – review & editing, Supervision, Project administration, Methodology, Conceptualization. **Beverley M. Essue:** Writing – review & editing, Supervision, Methodology, Conceptualization. **Sonica Singhal:** Writing – review & editing, Writing – original draft, Supervision, Methodology, Conceptualization. **Carlos Quiñonez:** Writing – review & editing, Supervision, Methodology, Conceptualization.

## Declaration of competing interest

The authors declare that they have no known competing financial interests or personal relationships that could have appeared to influence the work reported in this paper.
