# Supplementary material for: Oral health care’s contribution to catastrophic spending in Canada: a descriptive study
Source: Health Policy Open. 2025 Nov 20;10:100155. doi: 10.1016/j.hpopen.2025.100155 (PMC12682045; doi:10.1016/j.hpopen.2025.100155)
Supplement: Supplementary Data 1 [file mmc1.docx]

**Supplementary material**

**Oral Health Care’s Contribution to Catastrophic Spending in Canada: A Descriptive Study**

Contents

[**Table S1.** Summary of Survey of Household Spending Sample Selection (interview and diary) for households in the territories of Canada. 3](#_Toc210660682)

[**Table S2.** Summary of Survey of Household Spending Sample Selection (interview and diary) for households in the provinces of Canada. 4](#_Toc210660683)

[**File S1.** Household spending items recorded in the Survey of Household Spending 2010-2019 5](#_Toc210660684)

[**Table S3**. Definition of health care services paid out-of-pocket and COICOP 2018 comparison 7](#_Toc210660685)

[**Table S4**. Number and proportion of households spending out-of-pocket for oral health care and those with private supplementary insurance including oral health coverage 8](#_Toc210660686)

[**Table S5.** Number of households experiencing catastrophic health expenditure from 2010-2019 in Canada for people and households 9](#_Toc210660687)

[**Table S6.** Households in Canada organized by no out-of-pocket spending, and risk of impoverishment, impoverished, and further impoverished from out-of-pocket spending 10](#_Toc210660688)

[**File S2.** Households with catastrophic health spending by private supplementary insurance including oral health coverage and across income quintiles 11](#_Toc210660689)

[**Figure S1.** Catastrophic health expenditure in Canada by provinces and different catastrophic health expenditure methodology from 2010-2019. 14](#_Toc210660690)

[**Figure S2**. Share out-of-pocket spending among provincial households, those experiencing catastrophic health expenditure 15](#_Toc210660691)

[**Figure S3.** Share of health care out-of-pocket spending by income quintiles among households in Canada and those experiencing catastrophic health expenditure 16](#_Toc210660692)

[**Table S7.** Households who spend out-of-pocket on health care services across income quintiles, and among those facing catastrophic health expenditure 17](#_Toc210660693)

[**Figure S4**. Share out-of-pocket among household experiencing catastrophic health expenditure in 2010-2015 vs 2016-2019 18](#_Toc210660694)

[**Figure S5**. Share out-of-pocket among household experiencing catastrophic health expenditure by private supplementary insurance including oral health coverage 19](#_Toc210660695)

[**Figure S6**. Share out-of-pocket among household experiencing catastrophic health expenditure (WHO/Europe 40% capacity-to-pay approach) by private supplementary insurance including oral health coverage across provinces 20](#_Toc210660696)

[**Table S8**. Households who spend out-of-pocket on health care services across income quintiles, and last available year by having or not private supplementary insurance including oral health coverage 21](#_Toc210660697)

[**Table S9**. Households experiencing catastrophic health expenditure by private supplementary insurance including oral health coverage across health care services, income quintiles 22](#_Toc210660698)

**Catastrophic health expenditure budget share approach**

The budget share defines a household’s financial resource as either total income or consumption, thereby assuming a household can make use of their entire budget for health care spending [1]. Previous studies have favoured consumption over income as consumption is suggested to fluctuate less over time, although some authors recommend using income for high-income countries as households are more likely to pay using savings than loans [2].

As such, we decided to compare our WHO/Europe capacity-to-pay estimates using the budget share approach with both income and consumption in the denominator. Following the catastrophic health expenditure literature, we employed a 10% threshold for both income and consumption budget share approaches.

Total household income was defined as before-tax household income, including employment income, government transfers, retirement pensions, investment income, and other sources of income (e.g., scholarships, support payments, etc.).

Total household consumption was defined as household consumption on food, tobacco, alcohol, clothing and accessories, housing, household furnishing and equipment, health care, transportation, household operations, recreation, reading materials, education, personal care, and miscellaneous expenditure. Please see File S1 for further details on total household consumption.

For further information see:

[1] Nguyen HA, Ahmed S, Turner HC. Overview of the main methods used for estimating catastrophic health expenditure. Cost Eff Resour Alloc 2023;21:50. https://doi.org/10.1186/s12962-023-00457-5.

[2] Wagstaff A. Measuring catastrophic medical expenditures: Reflections on three issues. Health Econ 2019;28:765–81. https://doi.org/10.1002/hec.3881.

**Table S1.** Summary of Survey of Household Spending Sample Selection (interview and diary) for households in the territories of Canada.

|  | **Interview** | | | **Diary** | | |
| --- | --- | --- | --- | --- | --- | --- |
| **Year** | **Eligible** | **Respondents** | **Response rate** | **Eligible** | **Respondents** | **Response rate** |
| 2010 | NA | NA | NA | NA | NA | NA |
| 2011 | NA | NA | NA | NA | NA | NA |
| 2012 | 1,032 | 652 | 63.2% | NA | NA | NA |
| 2013 | NA | NA | NA | NA | NA | NA |
| 2014 | NA | NA | NA | NA | NA | NA |
| 2015 | 767 | 505 | 65.8% | 767 | 296 | 38.6% |
| 2016 | NA | NA | NA | NA | NA | NA |
| 2017 | 929 | 598 | 64.4% | 929 | 313 | 33.7% |
| 2019 | 937 | 590 | 63.0% | 937 | 364 | 38.8% |

*Sources:* Statistics Canada. User Guide for the survey of household spending, 2010. Ottawa: Statistics Canada = Statistique Canada; 2010; Statistics Canada. User Guide for the survey of household spending, 2011. Ottawa: Statistics Canada = Statistique Canada; 2011; Statistics Canada. User Guide for the survey of household spending, 2012. Ottawa: Statistics Canada = Statistique Canada; 2012; Statistics Canada. User Guide for the survey of household spending, 2013. Ottawa: Statistics Canada = Statistique Canada; 2013; Statistics Canada. User Guide for the survey of household spending, 2014. Ottawa: Statistics Canada = Statistique Canada; 2014; Statistics Canada. User Guide for the survey of household spending, 2015. Ottawa: Statistics Canada = Statistique Canada; 2015; Statistics Canada. User Guide for the survey of household spending, 2016. Ottawa: Statistics Canada = Statistique Canada; 2016. Statistics Canada. User Guide for the survey of household spending, 2017. Ottawa: Statistics Canada = Statistique Canada; 2017; Statistics Canada. User Guide for the survey of household spending, 2019. Ottawa: Statistics Canada = Statistique Canada; 2019.

NA: Not applicable

**Table S2.** Summary of Survey of Household Spending Sample Selection (interview and diary) for households in the provinces of Canada.

|  | **Interview** | | | **Diary** | | | |
| --- | --- | --- | --- | --- | --- | --- | --- |
| **Year** | **Eligible** | **Respondents** | **Response rate** | **Eligible** | **Respondents** | **Response rate** | **Missing from interview sample** |
| 2010* | 19,541 | 13,075 | 66.9% | 19,541 | 9,062 | 46.4% | 30.7% |
| 2011* | 17,873 | 11,746 | 65.7% | 17,873 | 7,661 | 42.9% | 34.8% |
| 2012 | 17,557 | 11,381 | 64.8% | 8,836 | 3,828 | 43.3% | 66.4% |
| 2013 | 17,389 | 11,686 | 67.2% | 8,782 | 4,048 | 46.1% | 65.4% |
| 2014 | 17,109 | 11,413 | 66.7% | 8,625 | 3,758 | 43.6% | 67.1% |
| 2015 | 17,603 | 10,723 | 60.9% | 12,571 | 5,010 | 39.9% | 53.3% |
| 2016 | 17,590 | 11,446 | 65.1% | 8,882 | 3,809 | 42.9% | 66.7% |
| 2017 | 17,792 | 11,894 | 66.9% | 8,948 | 3,699 | 41.3% | 68.9% |
| 2019* | 17,491 | 10,890 | 62.3% | 17,491 | 7,566 | 43.3% | 30.5% |

*Sources:* Statistics Canada. User Guide for the survey of household spending, 2010. Ottawa: Statistics Canada = Statistique Canada; 2010; Statistics Canada. User Guide for the survey of household spending, 2011. Ottawa: Statistics Canada = Statistique Canada; 2011; Statistics Canada. User Guide for the survey of household spending, 2012. Ottawa: Statistics Canada = Statistique Canada; 2012; Statistics Canada. User Guide for the survey of household spending, 2013. Ottawa: Statistics Canada = Statistique Canada; 2013; Statistics Canada. User Guide for the survey of household spending, 2014. Ottawa: Statistics Canada = Statistique Canada; 2014; Statistics Canada. User Guide for the survey of household spending, 2015. Ottawa: Statistics Canada = Statistique Canada; 2015; Statistics Canada. User Guide for the survey of household spending, 2016. Ottawa: Statistics Canada = Statistique Canada; 2016. Statistics Canada. User Guide for the survey of household spending, 2017. Ottawa: Statistics Canada = Statistique Canada; 2017; Statistics Canada. User Guide for the survey of household spending, 2019. Ottawa: Statistics Canada = Statistique Canada; 2019.

NA: Not applicable

*All households were contacted for the diary collection

**File S1.** Household spending items recorded in the Survey of Household Spending 2010-2019

Household spending items per year according to COICOP 2018 division

| ***Spending items*** | ***SHS item*** | ***COICOP***  ***Division*** | ***Survey of Household Spending year*** | | | | | | | | |
| --- | --- | --- | --- | --- | --- | --- | --- | --- | --- | --- | --- |
|  |  |  | ***2010*** | ***2011*** | ***2012*** | ***2013*** | ***2014*** | ***2015*** | ***2016*** | ***2017*** | ***2019*** |
| *Food consumption* | *FDREF_C* | 1.1; 1.2; 11.1 |  |  |  |  |  |  |  |  |  |
| *Tobacco, alcohol* | *TA018_C* | 2.1; 2.2; 2.3; 2.4 |  |  |  |  |  |  |  |  |  |
| *Clothing and accessories* | *CL030* | 3.1; 3.2 |  |  |  |  |  |  |  |  |  |
| *Housing* | *SH001* | 4.1; 4.3; 4.4; 4.5; 11.2 |  |  |  |  |  |  |  |  |  |
| *Household furnishing and equipment* | *HF001_C* | 5.1; 5.2; 5.3; 5.4; 5.5; 5.6 |  |  |  |  |  |  |  |  |  |
| *Health care* | *HC001_C* | 6.1; 6.2; 6.3; 6.4; 12.1 |  |  |  |  |  |  |  |  |  |
| *Transportation* | *TR001_C* | 7.1; 7.2; 7.3; 7.4 |  |  |  |  |  |  |  |  |  |
| *Household operations* | *HO001_C* | 8.1; 8.2; 8.3; 9.3 |  |  |  |  |  |  |  |  |  |
| *Recreation* | *RE001_C* | 9.1; 9.2; 9.4; 9.5; 9.6; 9.8 |  |  |  |  |  |  |  |  |  |
| *Reading materials* | *RO001_C* | 9.7 |  |  |  |  |  |  |  |  |  |
| *Education* | *ED002* | 10.1; 10.2; 10.3; 10.4; 10.5 |  |  |  |  |  |  |  |  |  |
| *Personal care* | *PC001_C* | 13.1; 13.2; 13.3; 13.9 |  |  |  |  |  |  |  |  |  |
| *Miscellaneous* | *ME001_C* | 12.2 |  |  |  |  |  |  |  |  |  |

Green= Spending items collected in Survey of Household Spending; COICOP: Classification of Individual Consumption According to Purpose 2018.

**More information on the SHS spending items, COICOP and catastrophic health expenditure**

To determine catastrophic health expenditure, we calculated the household’s total consumption and a basic needs line. The household’s total consumption included all items found in File S1, which are organized according to COICOP 2018. There are, however, some differences worth mentioning. For instance, compared to COICOP 2018, the SHS does not collect imputed rent (Division 04); instead, it collects information on mortgage payments, which are excluded from COICOP 2018. The WHO/Europe also suggests excluding imputed rent into the calculation of total expenditure, as how surveys deal with imputation techniques may differ per country. As the SHS includes a comprehensive list of expenditures for shelter, which includes mortgage, rent payers are not systematically seen as wealthier than non-rent payers. It is also worth mentioning that in our calculations, we were unable to obtain detailed information on food expenditure, as we did not use the SHS diary.

In terms of the basic needs line calculation, we followed the WHO/Europe’s catastrophic spending methodology. As such, for the basic needs line, we only included food consumption (COICOP section 1, which excludes alcoholic beverages and tobacco), rent consumption for the principal accommodation among renters (i.e., excluding mortgage payers) (COICOP section 4.1), and utilities consumption (COICOP section 4.4 and 4.5) among those paying utilities. Basic-needs line was constructed for each component of food, rent, and utilities expenditure per equivalent person (using OECD equivalence scale) and employing survey weights (at a national level). When households did not report spending on rent, we summed the rent and utilities needs-based line. Similarly, when households did not report on utilities, we summed the food and rent lines; and when households did not report on either rent or utilities, we only employed the food needs-based line. For households reporting on rent and utilities, the food, rent, and utilities needs-based line is summed.

The OECD equivalence scale calculation employed was:

Equivalent household size = 1 + 0.7 *(number of adults – 1) + 0.5 * (number of children under 13 years old)

We have excluded households reporting negative food expenditure, as it may suggest reporting error. The health care services spending categories and their categorization according to COICOP 2018 can be seen below in Table S3.

Compared to the WHO/Europe methodology, though, we have disaggregated our income quintiles based on equivalized income than consumption. We have made this choice as in Canada, the literature generally reports distribution of wealth based on income rather than consumption.

For more information on the WHO/Europe approach see:

*Thomson S, Cylus J, Al Tayara L, Martínez MG, García-Ramírez JA, Gregori MS, et al. Monitoring progress towards universal health coverage in Europe: a descriptive analysis of financial protection in 40 countries. Lancet Reg Health - Eur 2024;37.*

**Table S3**. Definition of health care services paid out-of-pocket and COICOP 2018 comparison

| **Health care service** | **Services** | **COICOP division** |
| --- | --- | --- |
| *Oral health care* | Dental care services (examinations, cleanings, fillings, extractions, x-rays, root canals, fittings and prescriptions for dentures, as well as orthodontics and periodontics). | 06.2.2 |
| *Medicines* | Prescribed medicines, drugs and pharmaceutical products | 06.1.1 |
| *Medical products* | 2010-2014: Prescription eye wear (e.g., contact lenses, eyeglasses, frames, insurance on lenses).  2015: Prescription eye wear, hearing aids and wheelchairs.  2016-2019: Prescription eye wear, and major health care equipment (including hearing aids, wheelchairs, three or four wheeled scooters, hospital beds, patient lifts and CPAP (Continuous Positive Airway Pressure) machines. | 06.1.2; 06.1.3 |
| *Outpatient care* | Home health care practitioners (e.g., nurses, attendant care, physiotherapists) excludes physician’s care. Includes other health care practitioners (e.g., nurses, physiotherapists, massage therapists, chiropractors, and naturopaths, etc.), health care by family doctors and specialists, and eye services (i.e., eye exams, eye surgery, etc.). | 06.2.1; 06.2.3 |
| *Diagnostic tests and other services* | Laboratory services, rental of medical equipment, ambulances, as well as weight control and quit-smoking programs | 06.4; 6.2.1.9 |
| *Inpatient and residential care* | Hospital care, nursing homes and other residential facilities including all charges (e.g., telephone, and TV rentals). | 06.3 |

COICOP: Classification of Individual Consumption According to Purpose 2018. Compared to WHO/Europe’s COICOP 2018 classification of health care services, we have included 06.2.3 in outpatient services (other outpatient care services) as some of these contain nursing home health care. The WHO/Europe places 06.2.3 in “diagnostic tests” category. We categorize “diagnostic tests” as COICOP’s 06.4 division “other health services”, which includes diagnostic imagining services and medical laboratory services, as well as patient emergency transportation services and emergency rescue. The other services included in “diagnostic tests” are best described in COICOP’s 6.2.1.9 “Other preventive services”.

**Table S4**. Number and proportion of households spending out-of-pocket for oral health care and those with private supplementary insurance including oral health coverage

| *Year* | *Households spending out-of-pocket for oral health care* | | | *Households with private* supplementary insurance including oral health coverage | | |
| --- | --- | --- | --- | --- | --- | --- |
|  | *n* | *%* | *95%CI* | *n* | *%* | *95%CI* |
| *2010* | 6.4 | 48.6% | [47.3—50.0] | 4.0 | 29.9% | [28.7—31.0] |
| *2011* | 6.4 | 47.8% | [46.4—49.2] | 4.2 | 31.0% | [29.8—32.3] |
| *2012* | 6.9 | 50.5% | [49.0—52.0] | 4.6 | 33.8% | [32.5—35.2] |
| *2013* | 7.0 | 50.9% | [49.5—52.3] | 4.6 | 33.1% | [31.8—34.4] |
| *2014* | 6.6 | 47.5% | [46.0—49.0] | 6.2 | 44.6% | [43.1—46.1] |
| *2015* | 6.9 | 48.9% | [47.5—50.3] | 6.4 | 45.8% | [44.4—47.2] |
| *2016* | 7.1 | 49.7% | [48.4—51.1] | 6.6 | 46.8% | [45.5—48.2] |
| *2017* | 7.2 | 50.6% | [49.2—51.9] | 6.5 | 45.8% | [44.5—47.1] |
| *2019* | 7.5 | 51.4% | [50.0—52.9] | 7.2 | 49.4% | [48.0—50.9] |

(n) Expressed in millions. All estimates are weighted; CI, confidence interval.

**Table S5.** Number of households/people experiencing catastrophic health expenditure from 2010-2019 in Canada

| *Year* | **Households (in millions)** | | | | | **People (in millions)** | | | | |
| --- | --- | --- | --- | --- | --- | --- | --- | --- | --- | --- |
|  | *Total sample* | *Catastrophic health expenditure* | | | | *Total sample* | *Catastrophic health expenditure* | | | |
|  |  | *CTP WHO/Europe* | | *BS 10% (C)* | *BS 10%(I)* |  | *CTP WHO/Europe* | | *BS 10% (C)* | *BS 10%(I)* |
|  |  | *40%* | *10%* | *10%* | *10%* |  | *40%* | *10%* | *10%* | *10%* |
| *2010* | 13.3 | 0.7 | 2.6 | 1.0 | 0.6 | 33.0 | 1.7 | 6.0 | 2.1 | 1.2 |
| *2011* | 13.4 | 0.7 | 2.7 | 1.0 | 0.6 | 33.3 | 1.7 | 6.0 | 2.1 | 1.2 |
| *2012* | 13.6 | 0.6 | 2.6 | 1.0 | 0.7 | 33.7 | 1.5 | 5.7 | 2.0 | 1.2 |
| *2013* | 13.8 | 0.6 | 2.5 | 0.9 | 0.6 | 34.1 | 1.5 | 5.6 | 1.8 | 1.0 |
| *2014* | 13.9 | 0.5 | 2.2 | 0.9 | 0.5 | 34.5 | 1.3 | 4.9 | 1.7 | 1.0 |
| *2015* | 14.0 | 0.5 | 2.2 | 0.9 | 0.6 | 34.8 | 1.4 | 5.1 | 1.8 | 1.0 |
| *2016* | 14.2 | 0.5 | 2.3 | 0.9 | 0.6 | 35.2 | 1.3 | 5.1 | 1.8 | 1.1 |
| *2017* | 14.3 | 0.4 | 2.2 | 0.8 | 0.6 | 35.5 | 1.0 | 4.7 | 1.6 | 1.1 |
| *2019* | 14.7 | 0.5 | 2.1 | 0.8 | 0.6 | 36.4 | 1.3 | 4.6 | 1.5 | 1.1 |
| *2010-2019* | 125.1 | 5.0 | 21.3 | 8.2 | 5.5 | 310.5 | 12.6 | 47.5 | 16.4 | 9.9 |

All estimates are weighted; CTP: Capacity to pay approach using normative spending on food, housing and utilities; BS: Basic share approach with consumption (C) as the denominator or income (I); Catastrophic health expenditure thresholds (40%, 10%).

**Table S6.** Households in Canada organized by no out-of-pocket spending, and risk of impoverishment, impoverished, and further impoverished from out-of-pocket spending

|  | **No out-of-pocket spending** | | **Not at risk of impoverishment** | | **At risk of impoverishment** | | **Impoverished** | | **Further impoverished** | |
| --- | --- | --- | --- | --- | --- | --- | --- | --- | --- | --- |
| **Year** | **n** | **%** | **n** | **%** | **n** | **%** | **n** | **%** | **n** | **%** |
| *Households* | |  |  |  |  |  |  |  |  |  |
| *2010* | 18.9 | 14.2 | 106.1 | 80.0 | 3.6 | 2.7 | 0.7 | 0.5 | 3.4 | 2.5 |
| *2011* | 19.7 | 14.6 | 106.8 | 79.4 | 3.7 | 2.8 | 0.8 | 0.6 | 3.4 | 2.5 |
| *2012* | 17.3 | 12.8 | 111.5 | 82.1 | 2.9 | 2.1 | 0.6 | 0.4 | 3.5 | 2.6 |
| *2013* | 17.7 | 12.9 | 112.6 | 81.8 | 3.2 | 2.3 | 0.7 | 0.5 | 3.5 | 2.5 |
| *2014* | 20.1 | 14.5 | 112.6 | 80.9 | 3.4 | 2.5 | 0.6 | 0.4 | 2.4 | 1.7 |
| *2015* | 21.1 | 15.0 | 112.7 | 80.2 | 3.7 | 2.6 | 0.3 | 0.2 | 2.7 | 1.9 |
| *2016* | 20.3 | 14.3 | 115.0 | 81.0 | 3.6 | 2.6 | 0.4 | 0.3 | 2.6 | 1.8 |
| *2017* | 19.2 | 13.5 | 118.2 | 82.8 | 3.0 | 2.1 | 0.4 | 0.2 | 1.9 | 1.3 |
| *2019* | 20.1 | 13.7 | 119.9 | 81.8 | 2.9 | 2.0 | 0.5 | 0.4 | 3.1 | 2.1 |
| *2010-2019* | 174.5 | 13.9 | 1015.3 | 81.1 | 30.1 | 2.4 | 5.0 | 0.4 | 26.4 | 2.1 |
| *People* | | | |  |  |  |  |  |  |  |
| *2010* | 40.8 | 12.4 | 268.8 | 81.4 | 8.8 | 2.7 | 1.5 | 0.4 | 10.2 | 3.1 |
| *2011* | 43.1 | 12.9 | 268.9 | 80.7 | 9.6 | 2.9 | 1.6 | 0.5 | 10.2 | 3.0 |
| *2012* | 37.0 | 11.0 | 281.8 | 83.6 | 7.1 | 2.1 | 1.3 | 0.4 | 9.9 | 2.9 |
| *2013* | 39.3 | 11.5 | 282.9 | 82.9 | 8.0 | 2.3 | 1.5 | 0.5 | 9.5 | 2.8 |
| *2014* | 44.7 | 13.0 | 282.0 | 81.8 | 8.8 | 2.5 | 1.5 | 0.4 | 7.8 | 2.3 |
| *2015* | 47.9 | 13.8 | 280.3 | 80.5 | 10.8 | 3.1 | 0.6 | 0.2 | 8.5 | 2.5 |
| *2016* | 47.9 | 13.6 | 286.9 | 81.6 | 7.8 | 2.2 | 1.1 | 0.3 | 7.9 | 2.3 |
| *2017* | 42.5 | 12.0 | 297.6 | 83.9 | 7.7 | 2.2 | 0.5 | 0.2 | 6.2 | 1.8 |
| *2019* | 43.5 | 11.9 | 302.4 | 83.0 | 8.3 | 2.3 | 1.0 | 0.3 | 9.3 | 2.5 |
| *2010-2019* | 386.6 | 12.5 | 2551.6 | 82.2 | 76.9 | 2.5 | 10.6 | 0.3 | 79.5 | 2.6 |

(n) in 100,000. Poverty lines constructed using the WHO/Europe capacity-to-pay approach at 40%.

**File S2.** Households with catastrophic health spending by private supplementary insurance including oral health coverage and across income quintiles

**
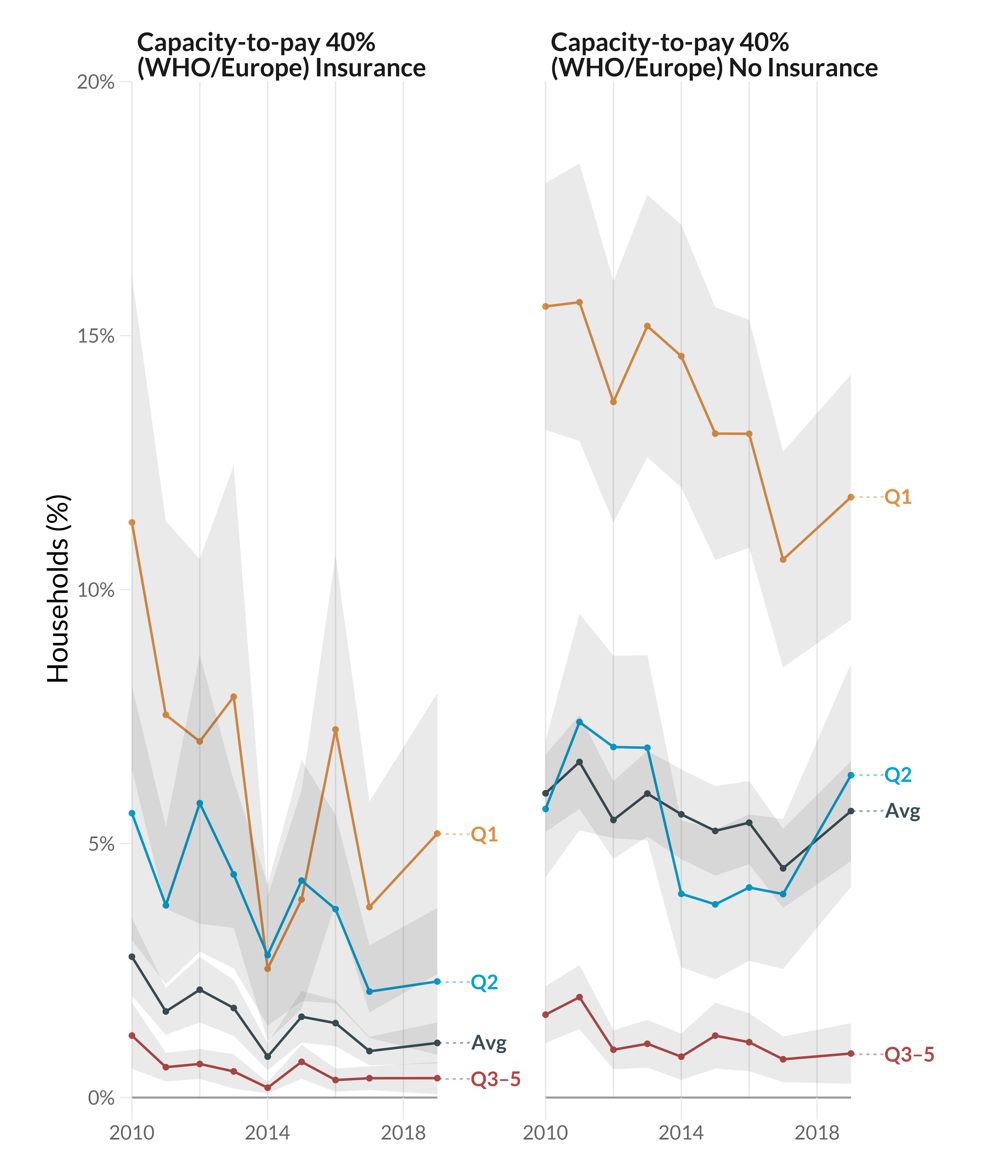
**

Capacity-to-pay approach defines household resources as normative food, rent, and utilities spending developed by the WHO Regional Office for Europe (WHO/Europe) at 40% threshold. Av, represents average.

Estimates for the other catastrophic methodologies below:

*With insurance*

|  |  | **Budget share 10% (consumption)** | | | **Budget share 10% (income)** | | |
| --- | --- | --- | --- | --- | --- | --- | --- |
| **Year** | **Quintile** | ***Percent*** | **95% CI** | | ***Percent*** | **95% CI** | |
| *2010* | Q1 | 11.29% | 6.6% | 16.0% | 14.65% | 9.5% | 19.8% |
| *2011* | Q1 | 5.01% | 2.5% | 7.6% | 16.88% | 11.1% | 22.6% |
| *2012* | Q1 | 6.99% | 3.3% | 10.6% | 18.15% | 11.6% | 24.7% |
| *2013* | Q1 | 6.50% | 3.5% | 9.5% | 20.12% | 13.3% | 27.0% |
| *2014* | Q1 | 5.84% | 0.8% | 10.9% | 14.79% | 6.8% | 22.8% |
| *2015* | Q1 | 4.08% | 1.7% | 6.4% | 13.93% | 9.2% | 18.7% |
| *2016* | Q1 | 5.04% | 1.8% | 8.2% | 13.22% | 8.4% | 18.0% |
| *2017* | Q1 | 4.71% | 2.0% | 7.4% | 17.12% | 11.6% | 22.6% |
| *2019* | Q1 | 4.50% | 2.4% | 6.6% | 14.48% | 10.0% | 19.0% |
| *2010* | Q2 | 10.01% | 7.1% | 12.9% | 5.45% | 3.1% | 7.8% |
| *2011* | Q2 | 6.21% | 4.3% | 8.1% | 3.16% | 1.8% | 4.5% |
| *2012* | Q2 | 8.57% | 5.4% | 11.8% | 5.94% | 3.0% | 8.8% |
| *2013* | Q2 | 7.88% | 5.3% | 10.4% | 2.61% | 1.4% | 3.8% |
| *2014* | Q2 | 9.13% | 6.3% | 12.0% | 2.74% | 1.6% | 3.9% |
| *2015* | Q2 | 6.93% | 4.4% | 9.5% | 3.79% | 2.2% | 5.4% |
| *2016* | Q2 | 5.42% | 3.4% | 7.5% | 3.80% | 2.0% | 5.6% |
| *2017* | Q2 | 5.40% | 3.6% | 7.2% | 3.96% | 2.4% | 5.6% |
| *2019* | Q2 | 4.45% | 2.7% | 6.2% | 2.64% | 1.5% | 3.8% |
| *2010* | Q3/5 | 6.0% | 4.9% | 7.2% | 1.58% | 1.0% | 2.2% |
| *2011* | Q3/5 | 6.8% | 5.5% | 8.1% | 1.71% | 1.0% | 2.4% |
| *2012* | Q3/5 | 6.7% | 5.4% | 8.0% | 1.96% | 1.1% | 2.8% |
| *2013* | Q3/5 | 5.7% | 4.6% | 6.9% | 1.51% | 0.9% | 2.1% |
| *2014* | Q3/5 | 4.7% | 3.7% | 5.6% | 1.06% | 0.6% | 1.5% |
| *2015* | Q3/5 | 6.0% | 5.0% | 7.1% | 1.72% | 1.1% | 2.3% |
| *2016* | Q3/5 | 4.5% | 3.7% | 5.4% | 1.14% | 0.7% | 1.6% |
| *2017* | Q3/5 | 5.0% | 4.1% | 5.9% | 1.52% | 1.0% | 2.0% |
| *2019* | Q3/5 | 4.2% | 3.3% | 5.1% | 1.21% | 0.8% | 1.6% |
| *2010* | Average | 7.12% | 6.1% | 8.2% | 3.30% | 2.5% | 4.1% |
| *2011* | Average | 6.56% | 5.5% | 7.6% | 3.18% | 2.4% | 4.0% |
| *2012* | Average | 7.03% | 5.9% | 8.2% | 4.20% | 3.2% | 5.2% |
| *2013* | Average | 6.17% | 5.2% | 7.2% | 3.13% | 2.4% | 3.9% |
| *2014* | Average | 5.47% | 4.5% | 6.4% | 2.51% | 1.7% | 3.4% |
| *2015* | Average | 5.99% | 5.1% | 6.9% | 3.20% | 2.5% | 3.9% |
| *2016* | Average | 4.73% | 4.0% | 5.5% | 2.60% | 2.0% | 3.2% |
| *2017* | Average | 4.64% | 5.8% | 7.1% | 2.76% | 5.2% | 6.5% |
| *2019* | Average | 6.42% | 5.8% | 7.1% | 5.87% | 5.2% | 6.5% |

*Without insurance*

|  |  | **Budget share 10% (consumption)** | | | **Budget share 10% (income)** | | |
| --- | --- | --- | --- | --- | --- | --- | --- |
| **Year** | **Quintile** | ***Percent*** | **95% CI** | | ***Percent*** | **95% CI** | |
| *2010* | Q1 | 8.14% | 6.3% | 10.0% | 12.23% | 10.0% | 14.5% |
| *2011* | Q1 | 8.56% | 6.5% | 10.7% | 12.78% | 10.2% | 15.4% |
| *2012* | Q1 | 6.64% | 5.1% | 8.2% | 12.42% | 10.1% | 14.7% |
| *2013* | Q1 | 6.46% | 4.9% | 8.0% | 11.73% | 9.5% | 13.9% |
| *2014* | Q1 | 8.40% | 6.3% | 10.5% | 11.73% | 9.2% | 14.2% |
| *2015* | Q1 | 6.14% | 4.6% | 7.7% | 10.35% | 8.1% | 12.6% |
| *2016* | Q1 | 6.57% | 4.9% | 8.3% | 9.16% | 7.2% | 11.2% |
| *2017* | Q1 | 6.50% | 4.9% | 8.1% | 12.46% | 10.1% | 14.8% |
| *2019* | Q1 | 6.71% | 5.0% | 8.4% | 12.25% | 10.0% | 14.5% |
| *2010* | Q2 | 12.37% | 10.3% | 14.4% | 6.16% | 4.6% | 7.8% |
| *2011* | Q2 | 11.87% | 9.5% | 14.2% | 5.45% | 3.8% | 7.1% |
| *2012* | Q2 | 11.33% | 8.8% | 13.8% | 5.53% | 4.0% | 7.1% |
| *2013* | Q2 | 10.15% | 8.2% | 12.1% | 4.41% | 3.2% | 5.6% |
| *2014* | Q2 | 9.58% | 7.4% | 11.8% | 4.43% | 3.0% | 5.8% |
| *2015* | Q2 | 9.43% | 7.2% | 11.6% | 5.53% | 3.8% | 7.3% |
| *2016* | Q2 | 10.80% | 8.4% | 13.2% | 6.63% | 4.6% | 8.6% |
| *2017* | Q2 | 8.74% | 6.8% | 10.7% | 5.20% | 3.6% | 6.8% |
| *2019* | Q2 | 8.56% | 6.4% | 10.7% | 6.26% | 4.3% | 8.2% |
| *2010* | Q3/5 | 6.4% | 5.5% | 7.4% | 1.63% | 1.1% | 2.2% |
| *2011* | Q3/5 | 6.5% | 5.4% | 7.5% | 1.79% | 1.2% | 2.4% |
| *2012* | Q3/5 | 6.2% | 5.1% | 7.4% | 1.49% | 0.9% | 2.1% |
| *2013* | Q3/5 | 6.2% | 5.0% | 7.4% | 1.73% | 0.9% | 2.6% |
| *2014* | Q3/5 | 4.5% | 3.3% | 5.6% | 1.35% | 0.7% | 2.1% |
| *2015* | Q3/5 | 5.8% | 4.6% | 6.9% | 1.72% | 1.1% | 2.4% |
| *2016* | Q3/5 | 6.7% | 5.4% | 8.1% | 2.48% | 1.6% | 3.4% |
| *2017* | Q3/5 | 5.8% | 4.6% | 7.0% | 1.62% | 1.0% | 2.2% |
| *2019* | Q3/5 | 4.5% | 3.3% | 5.7% | 1.39% | 0.8% | 2.0% |
| *2010* | Average | 8.15% | 7.3% | 9.0% | 5.26% | 4.5% | 6.0% |
| *2011* | Average | 8.15% | 7.2% | 9.1% | 5.36% | 4.5% | 6.2% |
| *2012* | Average | 7.46% | 6.5% | 8.4% | 5.13% | 4.4% | 5.9% |
| *2013* | Average | 7.11% | 6.3% | 8.0% | 4.91% | 4.1% | 5.7% |
| *2014* | Average | 6.81% | 5.8% | 7.8% | 5.10% | 4.2% | 6.0% |
| *2015* | Average | 6.71% | 5.8% | 7.6% | 5.10% | 4.3% | 5.9% |
| *2016* | Average | 7.66% | 6.7% | 8.6% | 5.48% | 4.6% | 6.4% |
| *2017* | Average | 6.42% | 5.8% | 7.1% | 5.87% | 5.2% | 6.5% |
| *2019* | Average | 6.42% | 5.8% | 7.1% | 5.87% | 5.2% | 6.5% |

**Figure S1.** Catastrophic health expenditure in Canada by provinces and different catastrophic health expenditure methodology from 2010-2019.


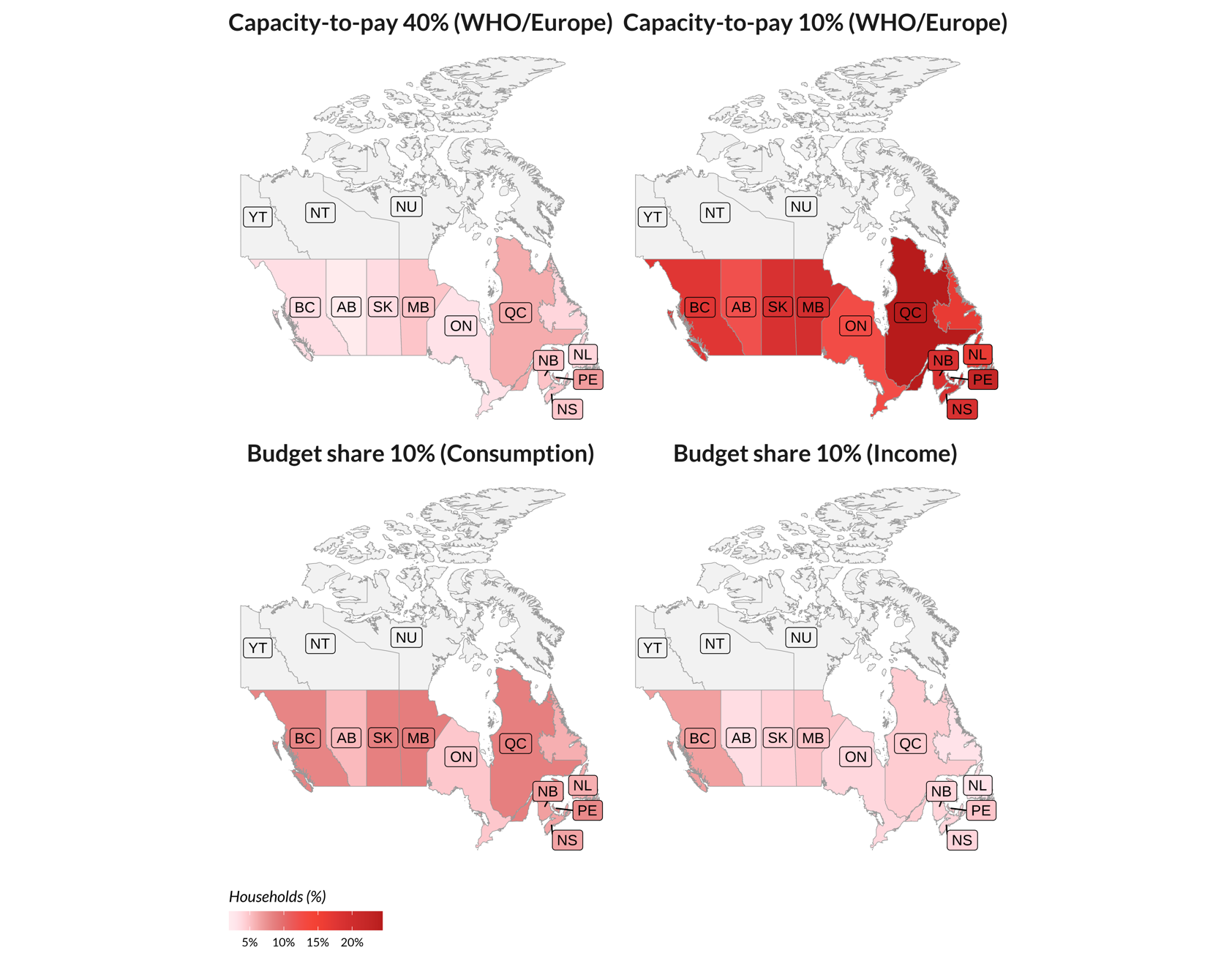


Capacity-to-pay approach defines household resources as total consumption net from normative food, rent, and utilities spending developed by the WHO Regional Office for Europe (WHO/Europe); Basic share approach defines household resources as either consumption or income. All catastrophic health expenditure thresholds are represented in % next to the capacity-to-pay and basic share methodology employed. BC, British Columbia; AB, Alberta; SK, Saskatchewan; MB, Manitoba; ON, Ontario; QC, Quebec; NB, New Brunswick; NL, Newfoundland & Labrador; PE, Prince Edward Island; NS, Nova Scotia. We don’t have data available for YT, Yukon; NT, Northwest Territories; and NU, Nunavut, the three territories in Canada.

**Figure S2**. Share out-of-pocket spending among provincial households, those experiencing catastrophic health expenditure


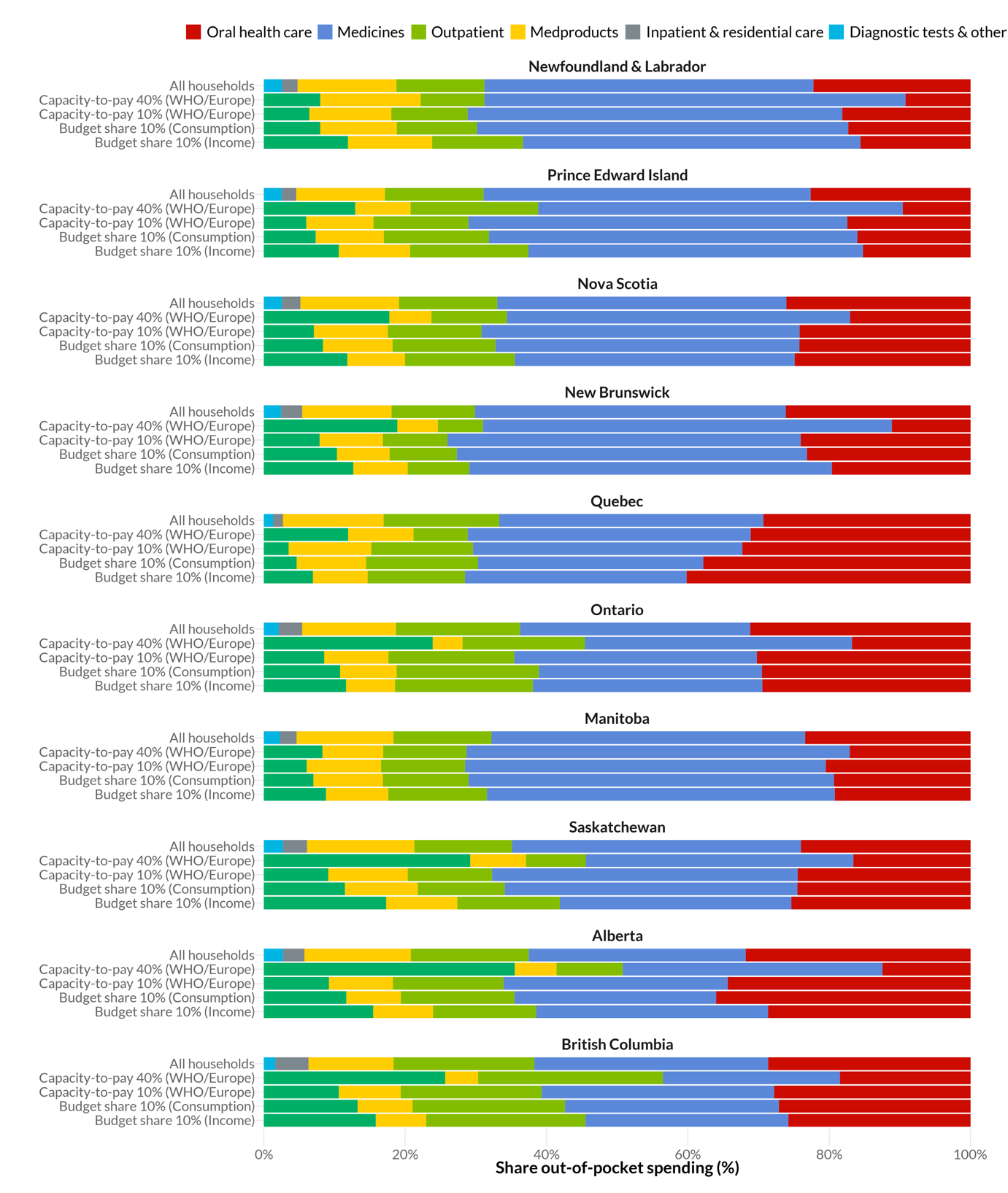
Capacity-to-pay approach defines household resources as total consumption net from normative food, rent, and utilities spending developed by the WHO Regional Office for Europe (WHO/Europe); Basic share approach defines household resources as either consumption or income. All catastrophic health expenditure thresholds are represented in % next to the capacity-to-pay and basic share methodology employed. Inpatient included hospital care, nursing homes and other residential facilities; diagnostic tests and other services included laboratory services, rental of medical equipment, ambulances, as well as weight control and quit-smoking programs.

**Figure S3.** Share of health care out-of-pocket spending by income quintiles among households in Canada and those experiencing catastrophic health expenditure


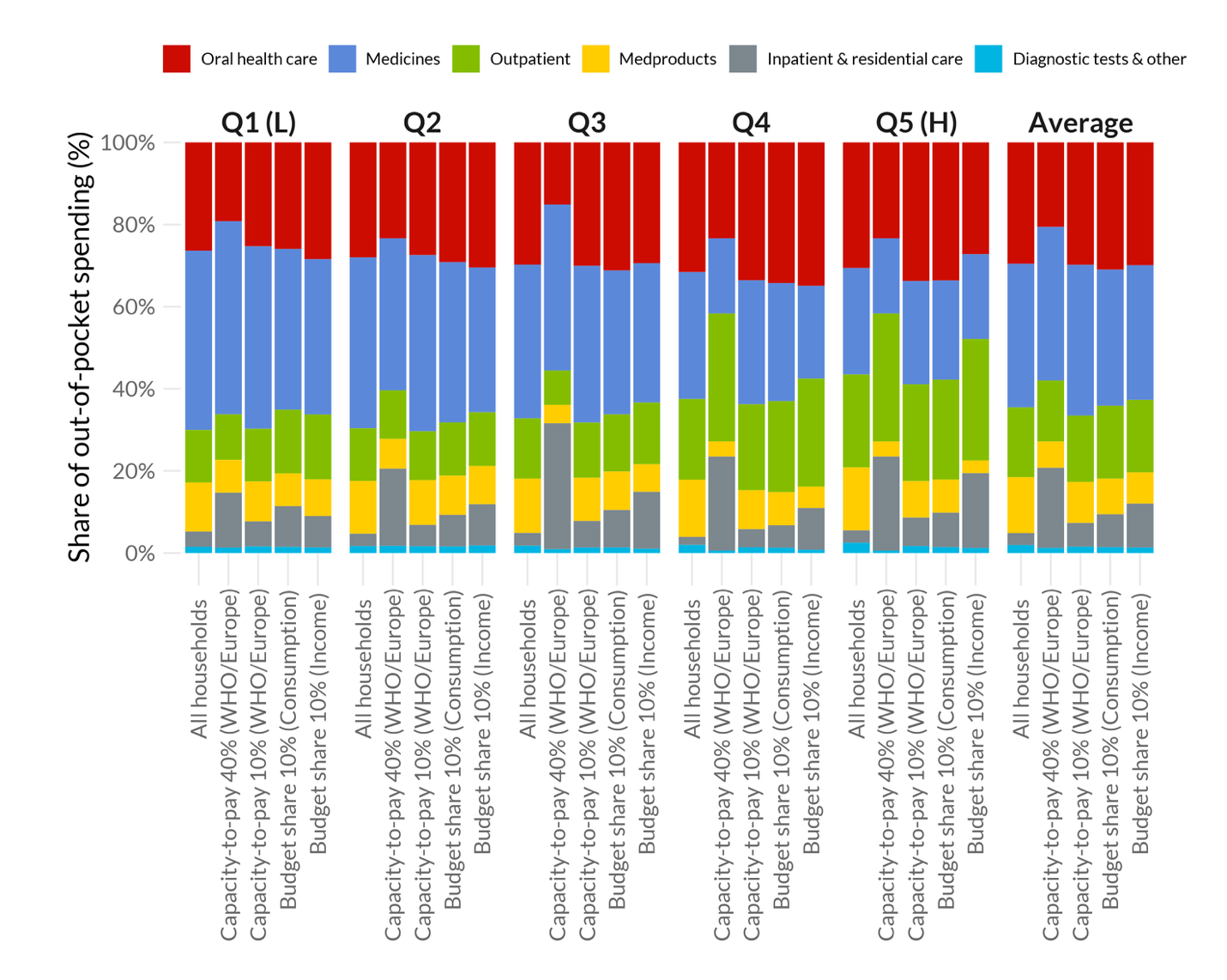
Capacity-to-pay approach defines household resources as normative food, rent, and utilities spending developed by the WHO Regional Office for Europe (WHO/Europe); Basic share approach defines household resources as either consumption or income. All catastrophic health expenditure thresholds are represented in % next to the capacity-to-pay and basic share methodology employed. Income quintiles (Q) ordered from lowest (L) to highest (H). Inpatient included hospital care, nursing homes and other residential facilities; diagnostic tests and other services included laboratory services, rental of medical equipment, ambulances, as well as weight control and quit-smoking programs.

**Table S7.** Households who spend out-of-pocket on health care services across income quintiles, and among those facing catastrophic health expenditure

|  | **Oral health care** | | **Medicines** | | **Outpatient care** | | **Medproducts** | | **Inpatient** | | **Diagnostics** | |
| --- | --- | --- | --- | --- | --- | --- | --- | --- | --- | --- | --- | --- |
| **Groups** | **n** | **%** | **n** | **%** | **n** | **%** | **n** | **%** | **n** | **%** | **n** | **%** |
| *General population* | | |  |  |  |  |  |  |  |  |  |  |
| *Q1* | 81.7 | 32.6% | 142.1 | 56.8% | 70.7 | 28.2% | 68.7 | 27.4% | 4.0 | 1.6% | 16.3 | 6.5% |
| *Q2* | 120.3 | 48.0% | 177.4 | 70.9% | 99.1 | 39.6% | 85.3 | 34.1% | 5.8 | 2.3% | 20.8 | 8.3% |
| *Q3* | 135.7 | 54.2% | 169.5 | 67.7% | 113.3 | 45.3% | 92.2 | 36.8% | 6.2 | 2.5% | 22.4 | 8.9% |
| *Q4* | 142.1 | 56.8% | 161.6 | 64.6% | 124.6 | 49.8% | 98.1 | 39.2% | 5.2 | 2.1% | 23.1 | 9.2% |
| *Q5* | 140.4 | 56.2% | 145.7 | 58.3% | 127.4 | 51.0% | 103.8 | 41.5% | 5.7 | 2.3% | 23.6 | 9.4% |
| *2019* | 75.4 | 51.4% | 93.7 | 63.9% | 67.3 | 46.0% | 55.4 | 37.8% | 2.8 | 1.9% | 11.5 | 7.9% |
| *2010/19* | 620.2 | 49.6% | 796.4 | 63.6% | 535.1 | 42.8% | 448.1 | 35.8% | 26.9 | 2.1% | 106.2 | 8.5% |
| *CTP 40%* | | |  |  |  |  |  |  |  |  |  |  |
| *Q1* | 9.2 | 29.6% | 23.0 | 74.4% | 8.7 | 28.2% | 9.4 | 30.3% | 0.7 | 11.0% | 3.4 | 2.3% |
| *Q2* | 5.1 | 41.7% | 10.6 | 86.1% | 4.9 | 39.8% | 4.3 | 35.1% | 0.9 | 10.4% | 1.3 | 7.3% |
| *Q3* | 1.9 | 47.3% | 3.5 | 88.0% | 1.5 | 39.0% | 1.3 | 32.0% | 0.4 | 12.4% | 0.5 | 10.5% |
| *Q4&5* | 1.6 | 66.7% | 1.9 | 79.3% | 1.0 | 42.3% | 0.9 | 36.2% | 0.5 | 15.9% | 0.4 | 20.9% |
| *2010/19* | 17.8 | 35.8% | 39.0 | 78.6% | 16.2 | 32.6% | 15.8 | 31.9% | 2.5 | 11.2% | 5.6 | 5.1% |
| *CTP 10%* | | |  |  |  |  |  |  |  |  |  |  |
| *Q1* | 28.6 | 40.8% | 57.3 | 81.7% | 26.5 | 37.9% | 26.4 | 37.6% | 2.2 | 10.0% | 7.0 | 3.1% |
| *Q2* | 33.9 | 54.9% | 55.9 | 90.6% | 30.4 | 49.2% | 27.4 | 44.4% | 2.5 | 11.0% | 6.8 | 4.1% |
| *Q3* | 25.0 | 67.9% | 32.9 | 89.6% | 21.1 | 57.4% | 17.8 | 48.5% | 1.9 | 13.1% | 4.8 | 5.2% |
| *Q4* | 20.4 | 76.6% | 23.6 | 88.6% | 18.6 | 69.6% | 13.9 | 52.0% | 1.4 | 14.7% | 3.9 | 5.2% |
| *Q5* | 14.4 | 80.3% | 15.1 | 84.0% | 12.1 | 67.4% | 9.8 | 54.5% | 1.4 | 16.2% | 2.9 | 7.7% |
| *2010/19* | 122.3 | 57.3% | 184.9 | 86.7% | 108.7 | 51.0% | 95.3 | 44.7% | 9.4 | 12.0% | 25.5 | 4.4% |
| *BS (CONS) 10%* | | |  |  |  |  |  |  |  |  |  |  |
| *Q1* | 9.8 | 56.6% | 15.7 | 91.3% | 8.8 | 50.8% | 7.5 | 43.6% | 1.2 | 14.0% | 2.4 | 7.0% |
| *Q2* | 15.1 | 65.3% | 21.6 | 93.5% | 13.4 | 58.0% | 11.9 | 51.8% | 1.5 | 13.5% | 3.1 | 6.6% |
| *Q3* | 12.8 | 76.0% | 15.3 | 91.3% | 10.3 | 61.3% | 8.5 | 50.6% | 1.3 | 15.6% | 2.6 | 8.0% |
| *Q4* | 11.8 | 80.6% | 13.2 | 90.0% | 10.9 | 73.8% | 7.4 | 50.2% | 0.9 | 15.7% | 2.3 | 6.0% |
| *Q5* | 9.2 | 85.0% | 9.3 | 86.1% | 7.4 | 68.2% | 6.1 | 56.0% | 1.2 | 16.8% | 1.8 | 11.0% |
| *2010/19* | 58.6 | 71.0% | 75.2 | 91.0% | 50.7 | 61.3% | 41.4 | 50.1% | 6.2 | 14.9% | 12.3 | 7.4% |
| *BS (INC) 10%* | | |  |  |  |  |  |  |  |  |  |  |
| *Q1* | 18.8 | 60.7% | 25.5 | 82.4% | 15.9 | 51.5% | 13.5 | 43.5% | 1.5 | 11.3% | 3.5 | 5.0% |
| *Q2* | 8.9 | 72.4% | 11.2 | 91.1% | 7.7 | 62.5% | 7.0 | 56.7% | 1.1 | 16.9% | 2.1 | 8.6% |
| *Q3* | 5.1 | 75.4% | 6.2 | 91.2% | 4.3 | 63.7% | 3.4 | 50.4% | 0.9 | 21.7% | 1.5 | 12.9% |
| *Q4* | 3.1 | 85.7% | 3.3 | 89.5% | 2.7 | 74.8% | 1.9 | 51.7% | 0.5 | 16.9% | 0.6 | 13.3% |
| *Q5* | 1.1 | 85.3% | 1.2 | 87.7% | 1.0 | 71.7% | 0.8 | 58.9% | 0.4 | 25.6% | 0.3 | 28.8% |
| *2010/19* | 37.0 | 67.3% | 47.3 | 86.1% | 31.6 | 57.5% | 26.5 | 48.2% | 4.3 | 14.6% | 8.0 | 7.9% |

(n) in 100,000 households. Capacity-to-pay approach (CTP) defines household resources as total consumption net of normative food, rent, and utilities spending developed by the WHO/Europe; Basic share (BS) approach defines household resources as either consumption (CONS) or income (INC). All catastrophic spending thresholds are represented in % next to the methodology employed. Income quintiles (Q) ordered from lowest (L) to highest (H). All estimates are weighted. Inpatient included hospital care, nursing homes and other residential facilities; diagnostic tests and other services included laboratory services, rental of medical equipment, ambulances, as well as weight control and quit-smoking programs.

**Figure S4**. Share out-of-pocket among household experiencing catastrophic health expenditure in 2010-2015 vs 2016-2019


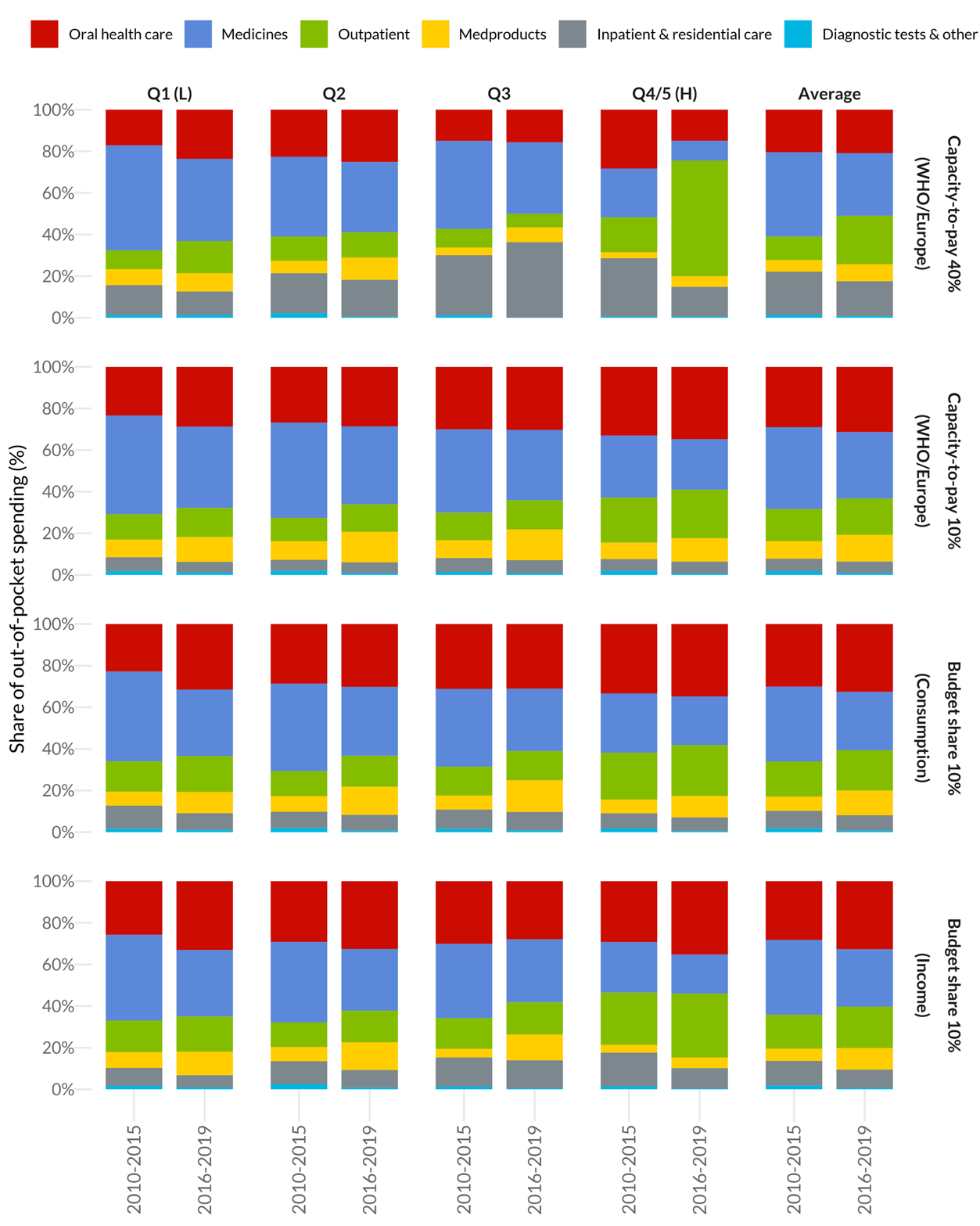


Capacity-to-pay approach defines household resources as total consumption net of normative food, rent, and utilities spending developed by the WHO Regional Office for Europe (WHO/Europe); Basic share approach defines household resources as either consumption or income. All catastrophic health expenditure thresholds are represented in % next to the capacity-to-pay and basic share methodology employed. Income quintiles (Q) ordered from lowest (L) to highest (H). Inpatient included hospital care, nursing homes and other residential facilities; diagnostic tests and other services included laboratory services, rental of medical equipment, ambulances, as well as weight control and quit-smoking programs.

**Figure S5**. Share out-of-pocket among household experiencing catastrophic health expenditure by private supplementary insurance including oral health coverage


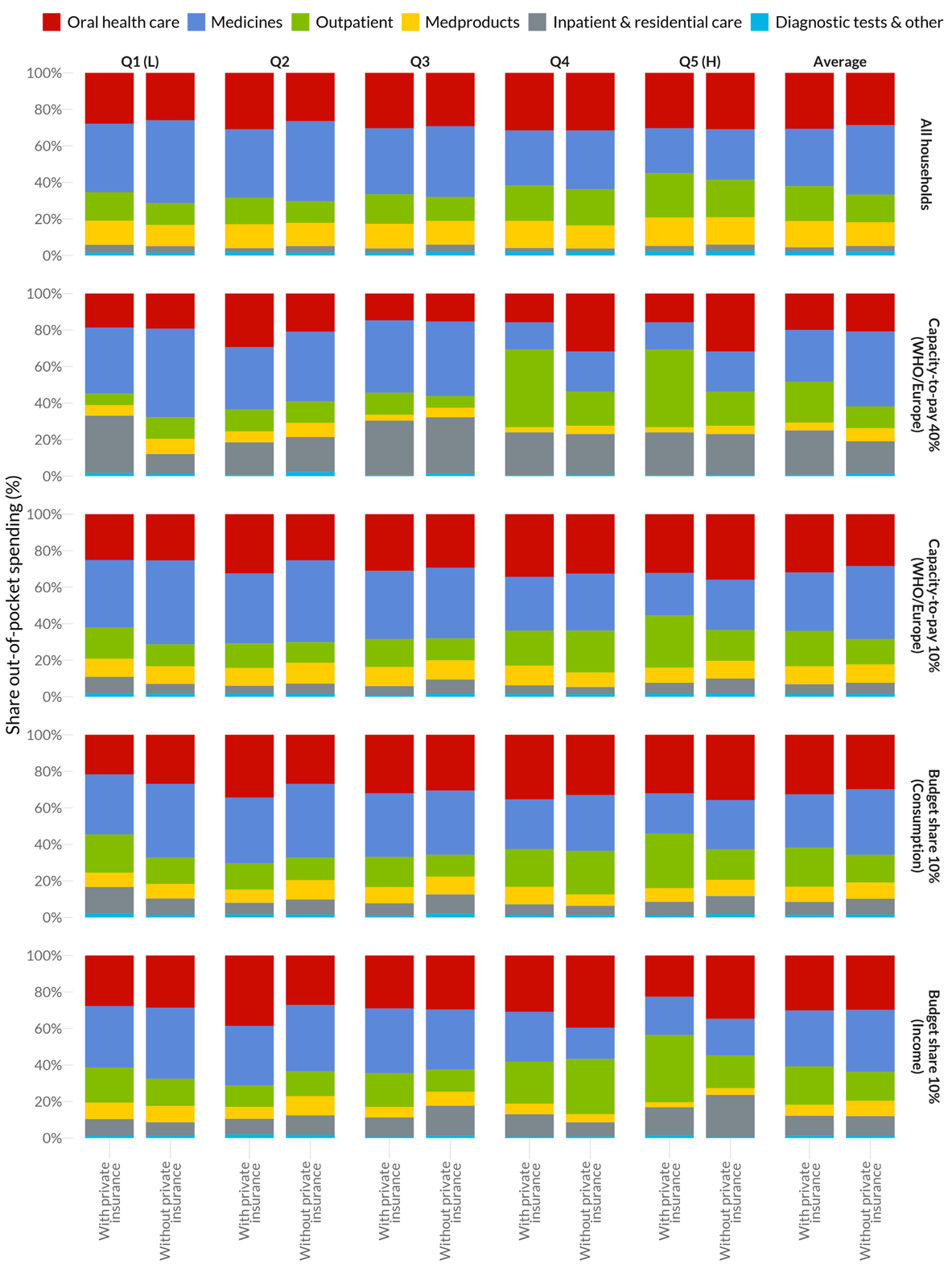


Capacity-to-pay approach defines household resources as total consumption net of normative food, rent, and utilities spending developed by the WHO Regional Office for Europe (WHO/Europe); Basic share approach defines household resources as either consumption or income. All catastrophic health expenditure thresholds are represented in % next to the capacity-to-pay and basic share methodology employed. Income quintiles (Q) ordered from lowest (L) to highest (H). Inpatient included hospital care, nursing homes and other residential facilities; diagnostic tests and other services included laboratory services, rental of medical equipment, ambulances, as well as weight control and quit-smoking programs.

**Figure S6**. Share out-of-pocket among household experiencing catastrophic health expenditure (WHO/Europe 40% capacity-to-pay approach) by private supplementary insurance including oral health coverage across provinces


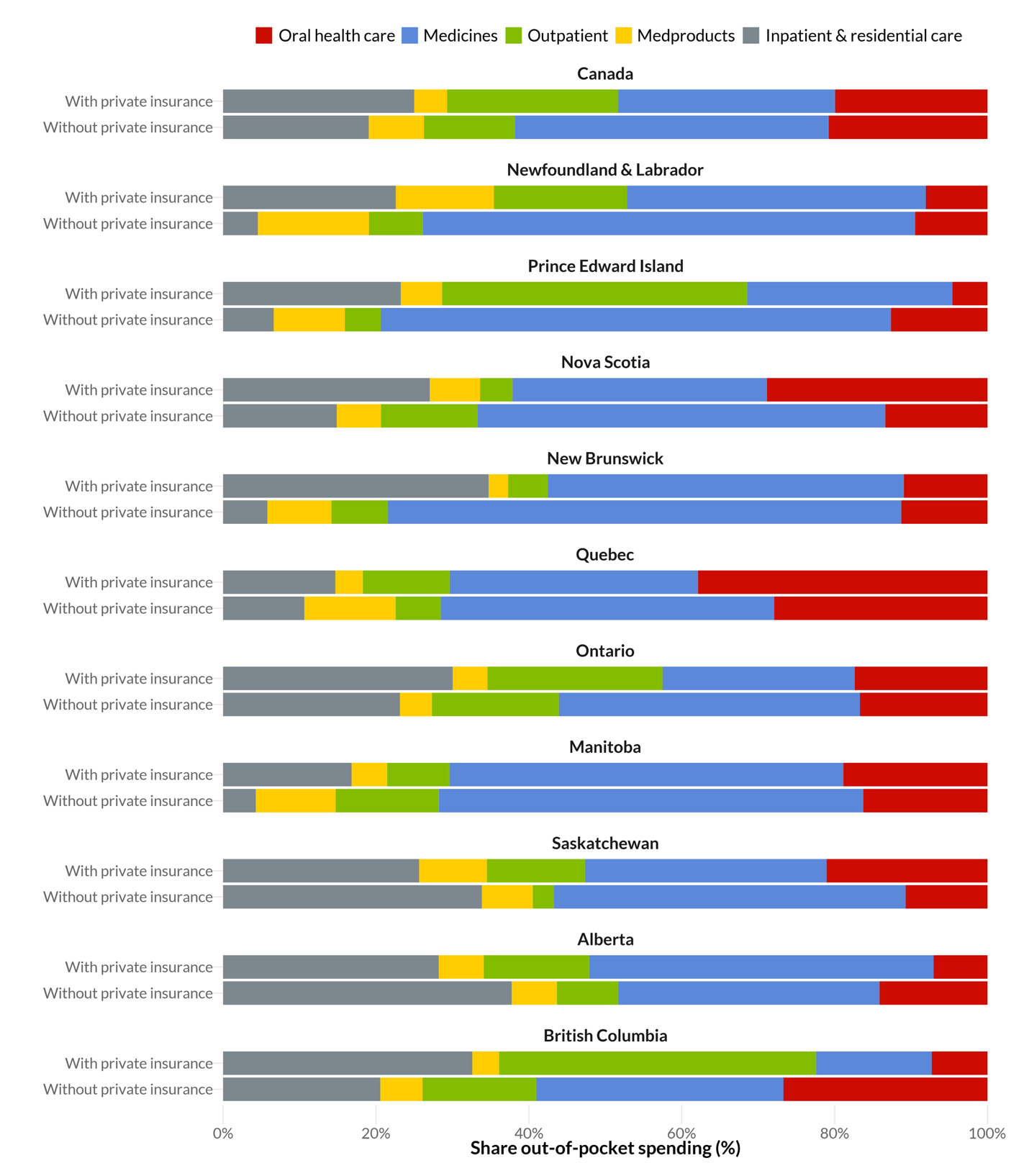


Inpatient included hospital care, nursing homes and other residential facilities; diagnostic tests and other services included laboratory services, rental of medical equipment, ambulances, as well as weight control and quit-smoking programs.

**Table S8**. Households who spend out-of-pocket on health care services across income quintiles, and last available year by having or not private supplementary insurance including oral health coverage

| **Income quintiles/ insurance** | **All health care services** | | **Oral health care** | | **Medicines** | | **Outpatient** | | **Medproducts** | | **Inpatients** | | **Diagnostics** | |
| --- | --- | --- | --- | --- | --- | --- | --- | --- | --- | --- | --- | --- | --- | --- |
|  | **n** | **%** | **n** | **%** | **n** | **%** | **n** | **%** | **n** | **%** | **n** | **%** | **n** | **%** |
| *Without private health insurance* | | |  |  |  |  |  |  |  |  |  |  |  |  |
| *Q1 (lowest)* | 208.0 | 83.1% | 61.7 | 30.0% | 114.9 | 55.0% | 53.6 | 26.0% | 53.4 | 26.0% | 3.2 | 2.0% | 13.3 | 6.0% |
| *Q2* | 167.7 | 67.0% | 73.0 | 44.0% | 118.0 | 70.0% | 60.2 | 36.0% | 53.3 | 32.0% | 3.9 | 2.0% | 12.7 | 8.0% |
| *Q3* | 133.7 | 53.4% | 64.4 | 48.0% | 86.3 | 65.0% | 53.4 | 40.0% | 45.8 | 34.0% | 3.1 | 2.0% | 11.5 | 9.0% |
| *Q4* | 118.3 | 47.3% | 59.2 | 50.0% | 70.2 | 59.0% | 50.4 | 43.0% | 41.3 | 35.0% | 2.1 | 2.0% | 9.9 | 8.0% |
| *Q5* | 120.1 | 48.0% | 59.2 | 49.0% | 64.0 | 53.0% | 53.9 | 45.0% | 45.3 | 38.0% | 2.6 | 2.0% | 9.9 | 8.0% |
| *2010–2019* | 747.8 | 59.8% | 317.7 | 42.0% | 453.3 | 61.0% | 271.5 | 36.0% | 239.1 | 32.0% | 14.8 | 2.0% | 57.3 | 8.0% |
| *2019* | 74.1 | 50.6% | 32.0 | 43.0% | 44.4 | 60.0% | 27.9 | 38.0% | 24.0 | 32.0% | 1.2 | 2.0% | 5.4 | 7.0% |
| *With private health insurance* | | |  |  |  |  |  |  |  |  |  |  |  |  |
| *Q1 (lowest)* | 42.3 | 16.9% | 19.9 | 47.0% | 27.3 | 64.0% | 17.1 | 40.0% | 15.3 | 36.0% | 0.8 | 2.0% | 3.0 | 7.0% |
| *Q2* | 82.6 | 33.0% | 47.2 | 57.0% | 59.5 | 72.0% | 38.9 | 47.0% | 32.0 | 39.0% | 1.9 | 2.0% | 8.1 | 10.0% |
| *Q3* | 116.5 | 46.6% | 71.3 | 61.0% | 83.1 | 71.0% | 59.8 | 51.0% | 46.4 | 40.0% | 3.1 | 3.0% | 10.9 | 9.0% |
| *Q4* | 132.0 | 52.7% | 82.8 | 63.0% | 91.4 | 69.0% | 74.3 | 56.0% | 56.8 | 43.0% | 3.1 | 2.0% | 13.2 | 10.0% |
| *Q5* | 130.0 | 52.0% | 81.2 | 62.0% | 81.7 | 63.0% | 73.5 | 57.0% | 58.4 | 45.0% | 3.1 | 2.0% | 13.6 | 10.0% |
| *2010–2019* | 503.5 | 40.2% | 302.5 | 60.0% | 343.0 | 68.0% | 263.6 | 52.0% | 209.0 | 42.0% | 12.1 | 2.0% | 48.8 | 10.0% |
| *2019* | 72.5 | 49.4% | 43.4 | 60.0% | 49.2 | 68.0% | 39.5 | 54.0% | 31.4 | 43.0% | 1.6 | 2.0% | 6.1 | 8.0% |

(n) in 100,000 households. Inpatient included hospital care, nursing homes and other residential facilities; diagnostic tests and other services included laboratory services, rental of medical equipment, ambulances, as well as weight control and quit-smoking programs.

**Table S9**. Households experiencing catastrophic health expenditure by private supplementary insurance including oral health coverage across health care services, income quintiles

|  | **Oral health care** | | **Medicines** | | **Outpatient** | | **Medproducts** | | **Inpatients** | | **Diagnostics** | |
| --- | --- | --- | --- | --- | --- | --- | --- | --- | --- | --- | --- | --- |
|  | **n** | **%** | **n** | **%** | **n** | **%** | **n** | **%** | **n** | **%** | **n** | **%** |
| *Without private health insurance* | | |  |  |  |  |  |  |  |  |  |  |
| *CTP 40%* | 13.9 | 32.9% | 32.7 | 77.7% | 12.8 | 30.3% | 13.2 | 31.2% | 1.8 | 11.0% | 4.7 | 4.4% |
| *Q1 (L)* | 8.1 | 28.4% | 21.0 | 73.9% | 7.7 | 27.0% | 8.5 | 29.8% | 0.6 | 10.7% | 3.0 | 2.1% |
| *Q2* | 3.6 | 39.0% | 8.0 | 86.2% | 3.4 | 37.1% | 3.3 | 35.4% | 0.7 | 10.3% | 1.0 | 7.5% |
| *Q3* | 1.1 | 39.5% | 2.4 | 88.3% | 1.0 | 35.6% | 0.8 | 29.5% | 0.3 | 15.2% | 0.4 | 9.4% |
| *Q4&5 (H)* | 1.1 | 64.8% | 1.3 | 77.0% | 0.7 | 38.7% | 0.6 | 35.0% | 0.3 | 14.9% | 0.3 | 18.1% |
| *CTP10%* | 77.3 | 51.1% | 129.9 | 85.9% | 68.3 | 45.2% | 62.4 | 41.3% | 6.4 | 10.9% | 16.4 | 4.2% |
| *Q1 (L)* | 24.0 | 38.9% | 50.1 | 81.3% | 21.9 | 35.6% | 22.2 | 36.0% | 1.9 | 9.9% | 6.1 | 3.0% |
| *Q2* | 23.4 | 51.1% | 41.8 | 91.3% | 21.3 | 46.4% | 19.5 | 42.6% | 2.0 | 10.2% | 4.7 | 4.3% |
| *Q3* | 14.0 | 64.2% | 19.6 | 89.5% | 11.6 | 53.1% | 10.0 | 45.6% | 1.2 | 12.9% | 2.8 | 5.3% |
| *Q4* | 9.4 | 72.2% | 11.4 | 87.5% | 8.5 | 65.2% | 5.9 | 45.7% | 0.7 | 12.8% | 1.7 | 5.2% |
| *Q5 (H)* | 6.5 | 73.7% | 7.0 | 79.4% | 5.0 | 57.2% | 4.7 | 53.4% | 0.7 | 13.3% | 1.2 | 8.2% |
| *BS (CONS) 10%* | 35.6 | 65.6% | 49.3 | 90.9% | 30.4 | 56.1% | 25.8 | 47.5% | 4.2 | 13.8% | 7.5 | 7.7% |
| *Q1 (L)* | 8.2 | 55.2% | 13.5 | 91.3% | 7.2 | 48.5% | 6.3 | 42.2% | 1.0 | 13.7% | 2.0 | 6.5% |
| *Q2* | 10.8 | 62.3% | 16.3 | 93.4% | 9.6 | 55.2% | 8.9 | 51.1% | 1.3 | 12.7% | 2.2 | 7.4% |
| *Q3* | 7.3 | 72.2% | 9.3 | 91.2% | 5.8 | 57.3% | 4.8 | 47.8% | 0.9 | 15.5% | 1.6 | 8.9% |
| *Q4* | 5.3 | 77.1% | 6.1 | 89.4% | 4.9 | 72.0% | 3.0 | 43.2% | 0.4 | 13.5% | 0.9 | 6.3% |
| *Q5 (H)* | 4.0 | 79.4% | 4.2 | 82.8% | 2.9 | 57.8% | 2.8 | 56.4% | 0.6 | 14.6% | 0.7 | 12.1% |
| *BS (INC) 10%* | 25.6 | 64.2% | 34.2 | 85.8% | 21.6 | 54.3% | 18.9 | 47.4% | 3.2 | 14.0% | 5.6 | 7.9% |
| *Q1 (L)* | 14.3 | 58.9% | 20.2 | 83.1% | 12.0 | 49.5% | 10.5 | 43.2% | 1.3 | 12.0% | 2.9 | 5.2% |
| *Q2* | 6.4 | 69.6% | 8.3 | 90.1% | 5.6 | 60.5% | 5.1 | 55.5% | 0.9 | 15.5% | 1.4 | 9.4% |
| *Q3* | 2.9 | 70.8% | 3.7 | 90.7% | 2.4 | 60.0% | 1.9 | 47.2% | 0.6 | 22.4% | 0.9 | 15.8% |
| *Q4* | 1.5 | 88.8% | 1.5 | 91.4% | 1.2 | 72.8% | 0.9 | 53.6% | 0.2 | 12.8% | 0.2 | 12.4% |
| *Q5 (H)* | 0.5 | 85.3% | 0.5 | 79.9% | 0.4 | 64.8% | 0.4 | 75.6% | 0.2 | 18.4% | 0.1 | 32.3% |
| *With private health insurance* | | |  |  |  |  |  |  |  |  |  |  |
| *CTP 40%* | 3.9 | 52.1% | 6.3 | 84.1% | 3.4 | 46.1% | 2.6 | 35.5% | 0.7 | 12.0% | 0.9 | 9.4% |
| *Q1 (L)* | 1.1 | 42.8% | 2.0 | 80.5% | 1.0 | 41.8% | 0.9 | 35.0% | 0.1 | 14.8% | 0.4 | 5.6% |
| *Q2* | 1.5 | 50.0% | 2.6 | 85.5% | 1.5 | 48.3% | 1.0 | 34.2% | 0.2 | 10.6% | 0.3 | 6.6% |
| *Q3* | 0.8 | 64.7% | 1.1 | 87.4% | 0.6 | 46.6% | 0.5 | 37.5% | 0.2 | 6.1% | 0.1 | 13.1% |
| *Q4&5 (H)* | 0.5 | 71.3% | 0.6 | 84.7% | 0.4 | 50.6% | 0.3 | 39.1% | 0.2 | 18.2% | 0.1 | 27.5% |
| *CTP10%* | 45.0 | 72.4% | 55.0 | 88.5% | 40.3 | 65.0% | 32.9 | 52.9% | 3.0 | 14.6% | 9.1 | 4.8% |
| *Q1 (L)* | 4.6 | 54.5% | 7.2 | 84.4% | 4.6 | 54.1% | 4.2 | 48.8% | 0.3 | 11.0% | 0.9 | 4.0% |
| *Q2* | 10.4 | 65.7% | 14.1 | 88.7% | 9.1 | 57.3% | 7.9 | 49.6% | 0.5 | 13.4% | 2.1 | 3.4% |
| *Q3* | 10.9 | 73.3% | 13.4 | 89.8% | 9.5 | 63.7% | 7.8 | 52.7% | 0.7 | 13.4% | 2.0 | 5.0% |
| *Q4* | 11.1 | 80.9% | 12.3 | 89.6% | 10.1 | 73.8% | 7.9 | 57.9% | 0.7 | 16.5% | 2.3 | 5.3% |
| *Q5 (H)* | 7.9 | 86.7% | 8.1 | 88.4% | 7.1 | 77.2% | 5.1 | 55.6% | 0.7 | 19.1% | 1.7 | 7.2% |
| *BS (CONS) 10%* | 23.0 | 81.1% | 25.9 | 91.2% | 20.2 | 71.2% | 15.6 | 55.0% | 2.0 | 17.0% | 4.8 | 6.9% |
| *Q1 (L)* | 1.6 | 65.5% | 2.2 | 91.6% | 1.6 | 64.6% | 1.3 | 52.1% | 0.2 | 16.0% | 0.4 | 10.1% |
| *Q2* | 4.2 | 74.8% | 5.3 | 93.8% | 3.8 | 66.6% | 3.0 | 53.8% | 0.2 | 16.2% | 0.9 | 4.2% |
| *Q3* | 5.4 | 81.7% | 6.1 | 91.5% | 4.5 | 67.4% | 3.6 | 54.8% | 0.4 | 15.9% | 1.1 | 6.5% |
| *Q4* | 6.6 | 83.6% | 7.1 | 90.6% | 5.9 | 75.4% | 4.4 | 56.3% | 0.5 | 17.6% | 1.4 | 5.8% |
| *Q5 (H)* | 5.2 | 89.8% | 5.2 | 89.1% | 4.5 | 77.2% | 3.2 | 55.7% | 0.6 | 18.6% | 1.1 | 10.1% |
| *BS (INC) 10%* | 11.4 | 75.5% | 13.1 | 86.7% | 10.0 | 65.8% | 7.6 | 50.3% | 1.2 | 16.1% | 2.4 | 7.8% |
| *Q1 (L)* | 4.4 | 67.0% | 5.3 | 79.8% | 3.9 | 58.9% | 2.9 | 44.3% | 0.3 | 8.8% | 0.6 | 4.2% |
| *Q2* | 2.4 | 80.7% | 2.9 | 94.4% | 2.1 | 68.4% | 1.8 | 60.2% | 0.2 | 21.2% | 0.6 | 6.1% |
| *Q3* | 2.3 | 82.1% | 2.5 | 92.0% | 1.9 | 69.1% | 1.5 | 54.9% | 0.2 | 20.6% | 0.6 | 8.8% |
| *Q4* | 1.7 | 83.0% | 1.7 | 88.0% | 1.5 | 76.4% | 1.0 | 50.1% | 0.3 | 20.3% | 0.4 | 14.2% |
| *Q5 (H)* | 0.6 | 85.3% | 0.7 | 93.8% | 0.6 | 77.0% | 0.3 | 46.0% | 0.2 | 31.2% | 0.2 | 26.1% |

(n) in 100,000 households; capacity-to-pay approach (CTP) defines household resources as total consumption net of normative food, rent, and utilities spending developed by the WHO Regional Office for Europe (WHO/Europe); Basic share (BS) approach defines household resources as either consumption (CONS) or income (INC). All catastrophic health expenditure thresholds are represented in % next to the capacity-to-pay and basic share methodology employed. Income quintiles (Q) ordered from lowest (L) to highest (H).
